# Impacts of COVID-19 pandemic on early life gut microbiome

**DOI:** 10.1080/19490976.2024.2443117

**Published:** 2024-12-31

**Authors:** Lin Zhang, Wenye Xu, Henry Y. H. Meng, Jessica Y. L. Ching, Yingzhi Liu, Shilan Wang, Shuai Yan, Ling Lin, Pui K. Cheong, Ka L. Ip, Ye Peng, Jie Zhu, Chun P. Cheung, Ting F. Leung, Agnes S. Y. Leung, Wing H. Tam, Tak Y. Leung, Paul K. S. Chan, Eugene B. Chang, David T. Rubin, Erika C. Claud, William K. K. Wu, Hein M. Tun, Francis K. L. Chan, Siew C. Ng

**Affiliations:** a Microbiota I-Center (MagIC), The Chinese University of Hong Kong, Hong Kong SAR, China; b Department of Anaesthesia and Intensive Care, Faculty of Medicine, The Chinese University of Hong Kong, Hong Kong SAR, China; c Department of Medicine and Therapeutics, Faculty of Medicine, The Chinese University of Hong Kong, Hong Kong SAR, China; d Jockey Club School of Public Health and Primary Care, Faculty of Medicine, The Chinese University of Hong Kong, Hong Kong SAR, China; e Department of Paediatrics, Faculty of Medicine, The Chinese University of Hong Kong, Hong Kong SAR, China; f Hong Kong Hub of Paediatric Excellence, The Chinese University of Hong Kong, Hong Kong SAR, China; g Department of Obstetrics and Gynaecology, Faculty of Medicine, The Chinese University of Hong Kong, Hong Kong SAR, China; h Department of Microbiology, Faculty of Medicine, The Chinese University of Hong Kong, Hong Kong SAR, China; i Department of Medicine, Section of Gastroenterology, Hepatology, and Nutrition, University of Chicago, Chicago, IL, USA; j Department of Pediatrics, Pritzker School of Medicine/Biological Sciences Division, University of Chicago, Chicago, IL, USA; k Institute of Digestive Disease and Li Ka Shing Institute of Health Sciences, Faculty of Medicine, The Chinese University of Hong Kong, Hong Kong SAR, China; l Center for Gut Microbiota Research, Faculty of Medicine, The Chinese University of Hong Kong, Hong Kong SAR, China

**Keywords:** COVID-19, pandemics, environment and public health, hygiene hypothesis, gut microbiome, early life

## Abstract

Increased hygiene and sanitation are theorized to predispose to developing atopic diseases, a process potentially mediated by the gut microbiome. We hypothesized that the gut microbiome maturation has been altered by COVID-19 lockdown measures during the first year of life, a critical period when environmental exposure shapes human microbiome development. The two large pre- and during-COVID-19 mother-baby pairs cohorts in the Greater Bay Area of China provided the unique opportunity to assess the effect of increased hygiene standards on early gut microbiome maturation. Our results showed that the gut microbiome diversity, composition, and developmental trajectory were significantly altered between pre- and during-COVID-19 cohorts. Functionally, there was decreased richness in both antimicrobial peptide resistance genes and antibiotic resistance genes in the during-COVID cohort. Specially, *Staphylococcus epidermidis* carried a lower copy number of fluoroquinolone and beta-lactam antibiotics resistance genes while *Klebsiella pneumoniae* possessed a higher copy number of fluoroquinolone antibiotic resistance genes in gut microbiota of infants born during the COVID-19 pandemic. Our study underscores the importance of considering the microbiome when evaluating hygiene measures and the need for future research to ascertain the role of the gut microbiome in disease development.

## Introduction

Since the beginning of the coronavirus (COVID-19) pandemic, stringent hygiene measures have been implemented to mitigate the spread of the virus. It has been hypothesized that this vigorous sanitation period may lead to atopic disease development due to poor gut microbiome stimulation.^1–3^ Epidemiological studies have shown that the prevalence of atopic dermatitis, allergic rhinitis or asthma has been on the rise between 1991 and 2006, especially in children of younger ages,^4^ with higher prevalence in affluent countries, although the prevalence is also rising in low to middle-income countries.^5–7^ The rise of atopic diseases has been linked to urbanization and increased hygiene standards.^8,9^ This has led to the development of the “hygiene hypothesis” and later the “microbiome hypothesis,” which implicates microorganism exposure in our living environment as a significant factor affecting the development of the immune system through its interactions with the human microbiome.^10,11^ Studies have suggested that environmental factors are the primary determinants affecting the gut microbiome and the immune system development.^12,13^ Cohort studies have shown that in urbanized environments, poor microorganism exposure in the early-life period negatively affects the diversity and development of the neonatal gut microbiome, which is associated with the future development of atopic diseases.^14,15^ Conversely, studies have shown that children living in rural environments in the early life period may be protected against atopic diseases.^16,17^ Such studies suggest that early life environmental exposure during critical periods of life may lead to long-term health detriments.^18,19^

Hong Kong has adopted stringent measures starting from the first cases of COVID-19, including border restrictions, social distancing measures, contact tracing, quarantine, and isolation.^20–22^ Furthermore, behavioral changes such as decreased travel, mask-wearing, use of alcohol-based sanitizers, and increased sterilization of the environment have also been practiced by the public.^21,22^ In this study, we hypothesize that these behaviors have affected the level of microbial exposure during birth and early infancy, which are the critical periods for microbiome establishment and development.^23,24^ Therefore, in this study, we aimed to characterize how the COVID-19 lockdown measures have affected the early-life gut microbiome in the first year of life.

## Results

### Study population characteristics

**Figure 1.**
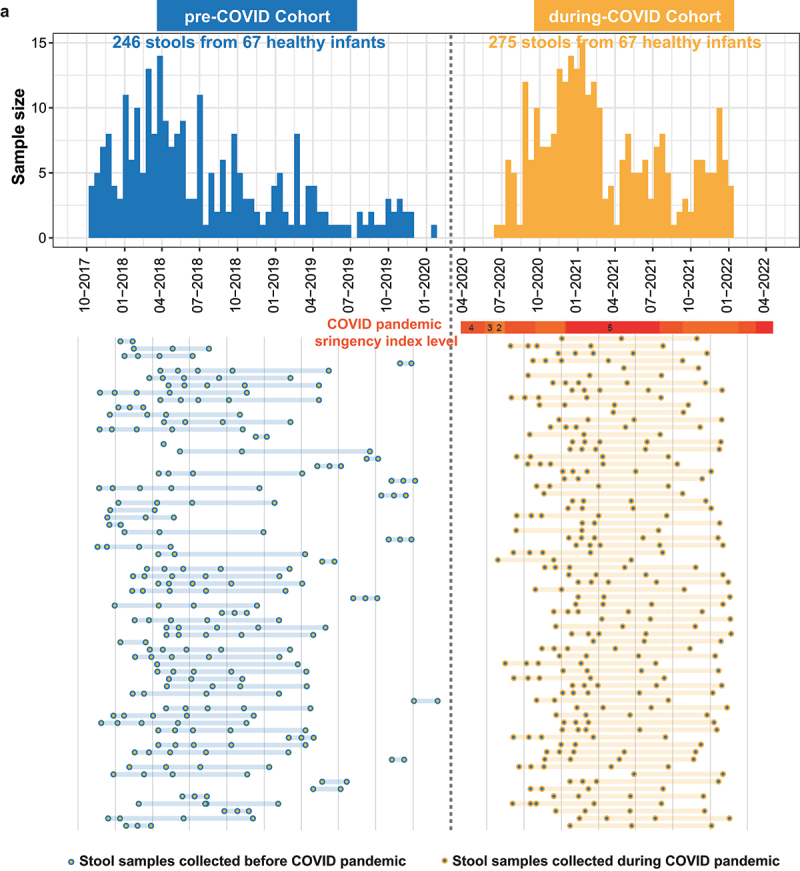
Sample collection summary and timeline. The histogram plot depicts the sample sizes of each time point after propensity matching by sex, delivery mode, and breastfeeding practice. The dots plot shows the collection timepoints of individual samples. Each dot indicates a sample, and each line indicates a subject. Red shaded bars delineate five levels of control-measure stringency in Hong Kong (level 1: <40; level 2 : 40–50; level 3: 50–60; level 4: 60–70; level 5: >70). Control-measure stringency applied in Hong Kong is based on the oxford COVID-19 government response tracker.

We included two longitudinal cohorts from Hong Kong: (1) the pre-COVID cohort (246 stools samples from 67 healthy infants collected before the COVID-19 pandemic during Oct 2017–Jan 2020); and (2) the during-COVID cohort (497 stools samples from 120 healthy infants collected during April 2020-Jan 2022). In addition, a total of 103 maternal stool samples (44 samples in the pre-COVID and 59 samples in the during-COVID cohort) were collected from the mothers in the 2nd to 3rd trimester. To minimize the difference in covariates between the two cohorts, we applied propensity matching (1:1) to adjust for sex, delivery mode, and breastfeeding practice for the infants. The study cohort demographics are summarized in Table 1 and Supplementary Table S1. There were no significant differences in demographic characteristics between the two cohorts after matching. The collection timepoints of individual samples and stringency index are depicted in Figure 1.

**Table 1.** Characteristics between pre-COVID and during-COVID cohort.

	Before propensity score matching			After propensity score matching		
	pre-COVID (*N* = 67)	during-COVID (*N* = 120)	p-value	pre-COVID (*N* = 67)	during-COVID (*N* = 67)	Adjusted p-value
**Sex**	··	··	0.70	··	··	0.73
Male	34 (50.7%)	56 (46.7%)	··	34 (50.7%)	31 (46.3%)	··
Female	33 (49.3%)	64 (53.3%)	··	33 (49.3%)	36 (53.7%)	··
**Delivery mode**	··	··	0.081	··	··	0.68
Vaginal delivery	54 (80.6%)	81 (67.5%)	··	54 (80.6%)	51 (76.1%)	··
Caesarean section	13 (19.4%)	39 (32.5%)	··	13 (19.4%)	16 (23.9%)	··
**Month1 Breastfeeding practice**	··	··	0.001	··	··	1
Almost exclusive breastfeeding*	11 (16.4%)	42 (35.0%)	··	11 (16.4%)	11 (16.4%)	··
Mixed feeding	49 (73.1%)	53 (44.2%)	··	49 (73.1%)	49 (73.1%)	··
Almost formula feeding^+^	7 (10.5%)	25 (20.8%)		7 (10.5%)	7 (10.5%)	
**Usage of intrapartum antibiotics**	··	··	0.09	··	··	0.72
Yes	24 (35.8%)	60 (50.0%)	··	24 (35.8%)	27 (40.3%)	··
No	43 (64.2%)	60 (50.5%)	··	43 (64.2%)	40 (59.7%)	··
**Oral Antibiotics within 3 months**	··	··	··	··	··	··
Month 1	5 (7.5%)	1 (0.8%)	0.02	5 (7.5%)	0	0.06
Month 2-3	5 (7.5%)	1 (0.8%)	0.02	5 (7.5%)	0	0.06
Month 6	0	2 (1.7%)	0.54	0	0	1
Month 12	1 (1.5%)	0	0.36	1 (1.5%)	0	1
**Furry pets**	··	··	1	··	··	0.68
Yes	13 (19.4%)	23 (19.2%)	··	13 (19.4%)	16 (23.9%)	··
No	54 (80.6%)	97 (80.8%)	··	54 (80.6%)	51 (76.1%)	··

Data are n (%) unless otherwise indicated.

^*^Almost exclusive breastfeeding: the proportion of breastfeeding higher than 90%.

^**+**^Almost formula feeding: the proportion of formula feeding higher than 90%.

### Microbial composition in infants born before and during COVID-19

**Figure 2.**
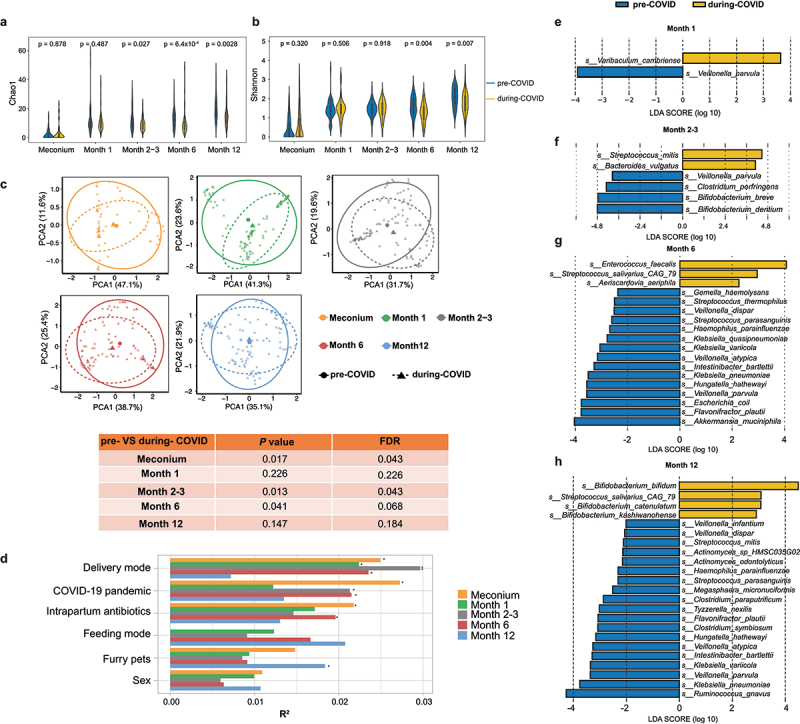
Gut microbial alpha diversity, beta diversity, and composition differed between pre-COVID and during-COVID. (a) Microbial richness was assessed by Chao1 index and (b) microbial diversity was assessed by Shannon index. *p* values were given by Wilcoxon’s rank-sum tests. (c) Principal component analysis (PCA) based on Bray-Curtis dissimilarity indicates significant differences in gut microbial community structure at birth, month 2-3, and month 6 between the pre-covid and during-covid. R^2^ and *p* values were given by permutational multivariate analysis of variance (PERMANOVA). Beta diversity was assessed with Bray-Curtis dissimilarity and was visualized by principal component analysis (PCA). (d) The bar plot depicts the effect size of host factors on gut microbiome at each timepoint. Effect size and statistical significance were determined via PERMANOVA. Asterisks indicate statistical significance with * *p* < 0.05. Differential bacterial species in infants between the two cohorts were detected by LEfSe at the age of (e) 1 month, (f) 2-3 months, (g) 6 months, and (h) 12 months.

All stool samples were subjected to shotgun metagenomics sequencing. In total, we sequenced 521 stool samples from 134 infants and 103 stool samples from mothers with an average of 6.789Gb sequence depth per sample. To rule out potential contamination, we used 6 negative controls in DNA extraction, library construction, and sequencing steps. Only unknown or unclassified taxa were detected in the negative controls after taxonomic annotation with MetaPhlAn3. Microbial Community Standard (ZymoBIOMICS™) was subject to repeated extraction, library construction, and metagenome sequencing to assess batch variation. Species-level relative abundances of positive controls revealed minimal variation between replicates (Supplementary Figure S1A). The sequencing depths of samples are depicted in Supplementary Figure S1B. We first examined changes in bacteria alpha diversity in infants between the two periods. Bacteria richness assessed by Chao1 index (Figure 2a) and observed species (Supplementary Figure S2A) did not differ at birth and 1 month old between the two cohorts. However, richness indices of the microbiome assessed at 2–3 months, 6 months and 12 months of age were significantly reduced in the during-COVID cohort compared with pre-COVID cohort (Chao1: *P*_M2–3_ = 0.027, *P*_M6_ = 6.4×10^−4^, *P*_M12_ = 0.0028; Observed species: *P*_M2–3_ = 0.031, *P*_M6_ = 8.7 × 10^−5^, *P*_M12_ = 1.6 × 10^−4^) (Figure 2a and Supplementary Figure S2A). Likewise, Shannon diversity was significantly reduced in the during-COVID cohort at 6 and 12 months of age (*P*_M6_ = 0.004, *P*_M12_ = 0.007) (Figure 2b) and phylogenetic diversity showed a decline from age 2 to 12 months when compared with the pre-COVID cohort (*P*_M2–3_ = 0.0011, *P*_M6_ = 1.7 × 10^−4^, *P*_M12_ = 2.0 × 10^−4^) (Supplementary Figure S2B). We used a Generalized Linear Model to assess the difference in alpha diversity between the cohorts after adjustment with sequence depths. Chao1 index and Shannon index remain higher in pre-COVID than during-COVID cohort after adjustment (data not shown). We also compared the development of alpha diversity index along the chronological age at sample collection timepoints in both groups. Microbial richness and diversity were persistently higher in the pre-COVID cohort than in the during-COVID cohort over the first year of life (Supplementary Figure S2C,D). Hong Kong implemented stringent measures from the onset of COVID-19. We next divided the during-COVID group into high stringency and low stringency subgroups based on whether the samples were collected under control measures with a stringency level higher than three.^21^ Notably, the observed species and Shannon diversity of gut microbiota were significantly lower in the high stringency group at 12 months of age compared to the low stringency group (Supplementary Figures S3A,B).

Microbial community structure, as assessed by the BrayCurtis dissimilarity metric, was significantly different between the pre- and during-COVID cohorts at birth, 2-3 months, and 6 months (Figure 2c, permutational multivariate analysis of variance (PERMANOVA): *P*_D0_ = 0.017, false discovery rate (FDR) *P*_FDR D0_ = 0.043; *P*_M2–3_ = 0.013, *P*_FDR M2–3_ = 0.043; *P*_M6_ = 0.041, *P*_FDR M6_ = 0.068). We next examined the impact of host factors on the infants’ gut microbiome composition. Among all host and environmental factors, delivery mode, COVID-19 pandemic, and intrapartum antibiotics were the top three factors that had the largest effect on early life gut microbiome composition (Figure 2d, PERMANOVA, FDR < 0.05). Differential species between the two groups were determined using Microbiome Multivariable Association with Linear Models (MaAsLin) to adjust for delivery mode, intrapartum antibiotics usage, household furry pets, and chronological age. Fifteen species were detected to be lower in the during-COVID cohort, including *Streptococcus thermophilus* and *Bifidobacterium breve* (Supplementary Table S2). We furthermore identified differential species between the two cohorts at each sample collection timepoint (Figure 2e–h, Supplementary Figure S4A-D) using linear discriminant analysis (LDA) effect size (LEfSe) and MaAsLin methods. No differential species were observed in meconium samples between the two cohorts. Interestingly, the abundance of two *Bifidobacterium* species, *Bifidobacterium breve* and *Bifidobacterium dentium*, were significantly reduced in the during-COVID cohort at 2-3 months after birth detected by LEfSe (Figure 2f) and MaAsLin (Supplementary Figure S4B, adjusted with delivery mode, intrapartum antibiotics usage, and household furry pets). Interestingly, the abundances of *Klebsiella pneumoniae* and *Klebsiella variicola* were decreased in the during-COVID cohort at months 6 and 12 when compared to the pre-COVID cohort. A sensitivity analysis that excluded the infants who were exposed to antibiotics was performed to investigate the difference in gut microbiota alpha diversity and composition between the two cohorts and the results were consistent (Supplementary Figure S5A-C).

### COVID-19 pandemic played the major determinant impacting the maternal gut microbiota composition

**Figure 3.**
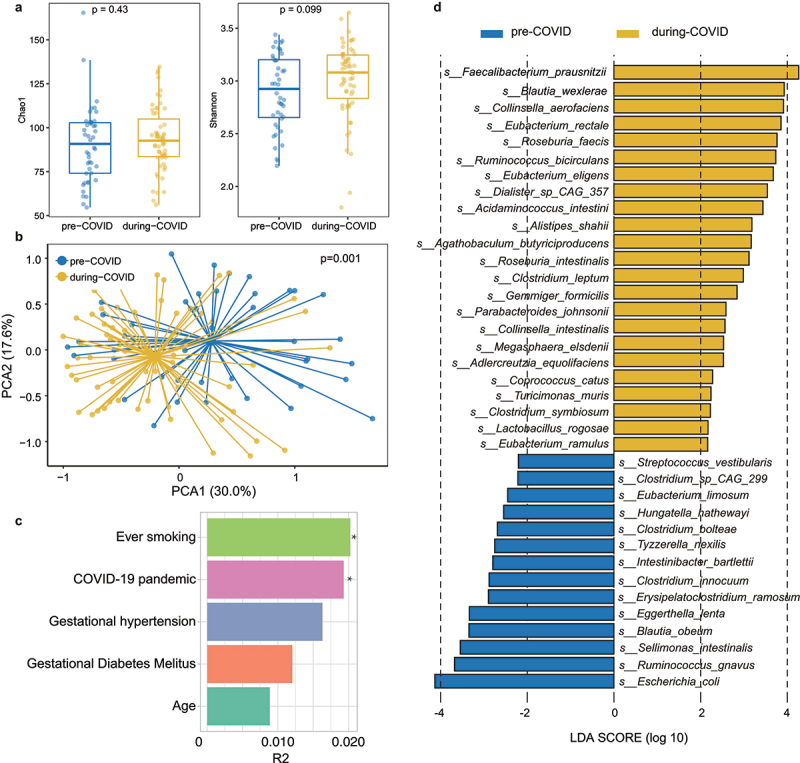
Difference in maternal gut microbiota diversity and composition between pre-COVID and during-COVID cohort (a) microbial richness was assessed by Chao1 index and microbial diversity was assessed by Shannon index. *p* values were given by Wilcoxon’s rank-sum tests. (b) Principal component analysis (PCA) based on Bray-Curtis dissimilarity indicated significant differences in gut microbial community structure. (c) The bar plot depicts the effect size of host factors on maternal gut microbiome. Effect size and statistical significance were determined via PERMANOVA. Asterisks indicate statistical significance with *p < 0.05. (d) Differential species in maternal stool between the two cohorts were determined by LEfSe.

We further performed the metagenomic analysis to determine if the maternal microbiome was significantly altered during the lockdown measures of COVID-19, and if this alteration had played a role in the changes in the infant gut microbiome between pre- and during-COVID cohort. A total of 103 maternal stool samples (44 samples in the pre-COVID and 59 samples in the during-COVID cohort) collected from the mothers in the 2^nd^ to 3^rd^ trimester were included. The demographics of mothers are summarized in Supplementary Table S3. Our results showed that maternal gut microbiome alpha diversity assessed by Chao 1 index and Shannon diversity did not differ in pre- and during-COVID cohorts (Figure 3a), although there was a significant change in overall gut microbiota composition (Figure 3b). Among all host and environmental factors, including age, smoking status, gestational hypertension, gestational diabetes mellitus, and the COVID-19 pandemic, we observed that COVID-19 lockdown measures and smoking status were the major determinants impacting the maternal gut microbiota composition (Figure 3c). We identified the differential species in maternal stool in pre- and during-COVID groups by LEfSe (Figure 3d) and compared them with the infant gut microbiome differential species. However, the maternal differential species did not overlap with the differential bacterial species in infant stools collected at month 1 or month 2-3 between pre-COVID and during-COVID cohorts (Figure 2e,f). We also examined the difference in mother-to-infant transmission between the two cohorts via PanPhlAn and StrainPhlAn. There were no significant differences in the strain-sharing rate between mother-infant pairs across the two cohorts (Supplementary Figure S6). Additionally, no species exhibited significant differences in transmission from mother to infant when comparing the pre-COVID and during-COVID cohorts (Supplementary Table S4). This may suggest that there are other factors aside from vertical transmission that account for the changes in the infant microbiome.

### COVID-19 pandemic altered the trajectory of gut microbial development in infants

**Figure 4.**
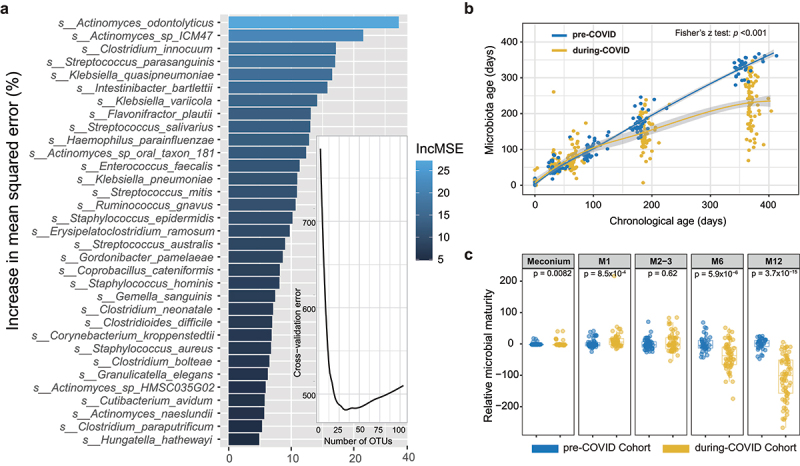
Gut microbial maturation altered in infants born during COVID-19 pandemic (a) bar plot shows the variable importance in the prediction model of estimated microbial age in healthy individuals. The minimal number of age-discriminatory species was determined by minimizing the cross-validation error. 33 age-discriminatory bacterial species were identified based on gut bacteria of healthy infants in pre-COVID cohort by random forest. (b) Development of estimated microbial age throughout infancy among infants pre- and during COVID-19 as modeled by loess regression. Gray areas represent 95% confidence intervals. (c) The relative microbial maturity was significantly increased in healthy infants in during-COVID cohort from birth to 1 month old whereas decreased dramatically at the age of 6 months and 12 months compared with pre-COVID cohort.

To evaluate gut microbial maturation, we developed a microbiome-age prediction model using Random Forest based on the gut microbiome species-level profiles of healthy infants from the pre-COVID cohort and identified 33 age-discriminatory species (Figure 4a). To assess the performance of the gut microbiome-age prediction model, we drew 1,000 bootstrap samples which were repeated 100 times. The mean absolute error (MAE, 95% CI) was 13.5 [13.4,13.6] days. We further correlated the estimated gut microbiota age with chronological age. The estimated gut microbiota age was strongly correlated with chronological age with a mean kendall’s tau coefficient of 87.9% [87.8%, 88.0%]. Using this prediction model, our data showed that the estimated microbial age of infants born during COVID-19 tended to be higher in the first 100 days but subsequently developed at a slower rate in comparison with those born pre-COVID-19 (Figure 4b). We then calculated the relative microbiota maturity (RMM) using an established formula that compares postnatal assembly (defined here as maturation) of an infant’s fecal microbiota relative to infants of similar chronologic age in the pre-COVID cohort.^25^ We found that the RMM was significantly increased in pre-COVID cohort from birth to 1 month old but decreased at the age of 6 months and 12 months as compared with the during-COVID cohort (Figure 4c). To adjust other confounding factors, we further compared the differences in gut microbial relative maturity between the pre-COVID and during-COVID cohorts, stratified by delivery mode, intrapartum antibiotic exposure, and the presence of household pets. Infants born during the COVID-19 pandemic still exhibited lower gut microbial relative maturity at the ages of 6 and 12 months compared to those born pre-COVID pandemic, even after stratification (Supplementary Figure S7A-C). Additionally, the gut microbial relative maturity in the during-COVID cohort remains lower than that of the pre-COVID cohort at the ages of 6 and 12 months after excluding those infants with antibiotic exposure (Supplementary Figure S7D).

### Alterations in microbial functional pathway in infants born during COVID-19

**Figure 5.**
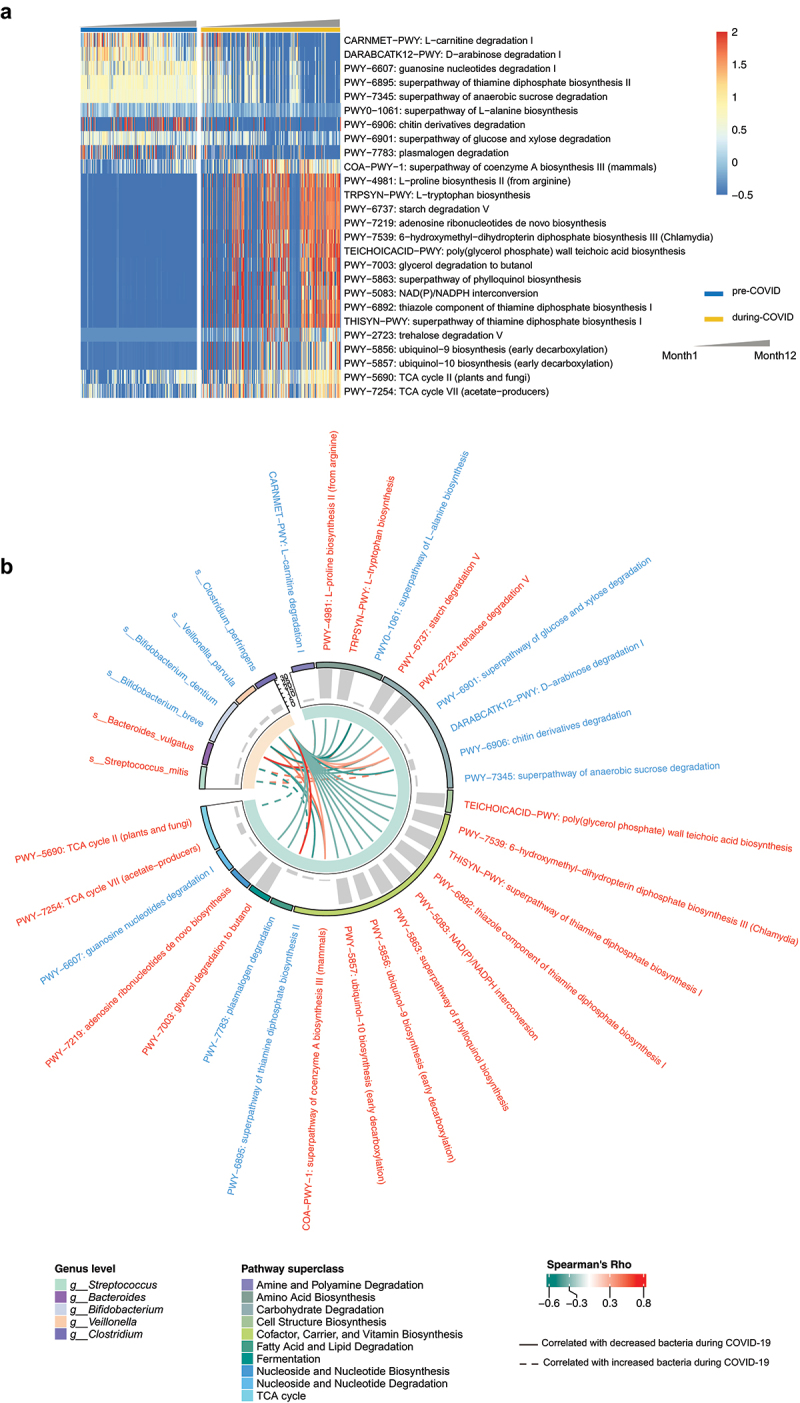
Differential microbial pathways between infants in pre-COVID and during-COVID cohort in the first year. (a) Heatmap depicts the differential pathways between the two cohorts. Differential pathways were determined by Deseq2 (all adjusted *p* < 0.05) and followed by adjustments for chronological age, delivery mode, intrapartum antibiotics usage, and household furry pets using MaAsLin (all adjusted *p* < 0.05). The color bar represents the row-normalized abundances of pathways. Samples were arranged by chronological age in ascending order. (b) Associations between differential species and pathways. Differential gut bacterial species were detected by LEfSe. The red letters indicate enriched bacterial species or pathways in the during-COVID cohort, and the blue letters represent decreased bacterial species or pathways in the during-COVID cohort. The gray bar indicates the log2-fold change of species or pathways between pre-COVID cohort and during-COVID cohort. Associations were calculated by HAIIA. Pairwise correlations between selected bacterial species and pathways markers with q-value <0.15 were shown.

Given that microbiota composition changed dramatically in early life during COVID-19, we next applied DESeq2 and MaAsLin to identify differential microbial pathways between the two groups. A total of 10 pathways were depleted, while 16 were enriched in the during-COVID cohort during the first year after adjusting for chronological age, intrapartum antibiotics usage, delivery mode, and having household furry pets (Figure 5a, Supplementary Table S5). Pathways related to vitamin biosynthesis (PWY-6895: superpathway of thiamine diphosphate biosynthesis II), sugar degradation (DARABCATK12-PWY: D-arabinose degradation I; PWY-7345: superpathway of anaerobic sucrose degradation) and purine nucleotide degradation (PWY-6607: guanosine nucleotides degradation I) were the top 4 functional pathways negatively correlated with the during-COVID cohort after adjustment. Among pathways enriched in the during-COVID cohort, there were three pathways associated with microbial energy generation, namely PWY-5690: tricarboxylic acid (TCA) cycle II, PWY-7254: TCA cycle VII, and PWY-5083: NADP/NADPH interconversion (Figure 5a, Supplementary Table S5).

Four bacteria species, *Bifidobacterium breve*, *Bifidobacterium dentium*, *Veillonella parvula*, and *Clostridium perfringens*, depleted in infants during the COVID-19 pandemic, were identified to be associated with most of the altered microbial function. (Figure 5b, Supplementary Table S6). Among them, *Bifidobacterium breve* was identified to be significantly related to L-alanine biosynthesis (Figure 5b, and Supplementary Table S6). No significant correlation was found between the specific increased species during COVID-19 and pathways associated with microbial energy generation (Supplementary Table S6). These results provide evidence that the depletion of specific beneficial species in infants born during COVID-19 is associated with functional changes in the gut microbiome.

### COVID-19 pandemic dampened the gut microbiota adaptation to antimicrobial peptide

**Figure 6.**
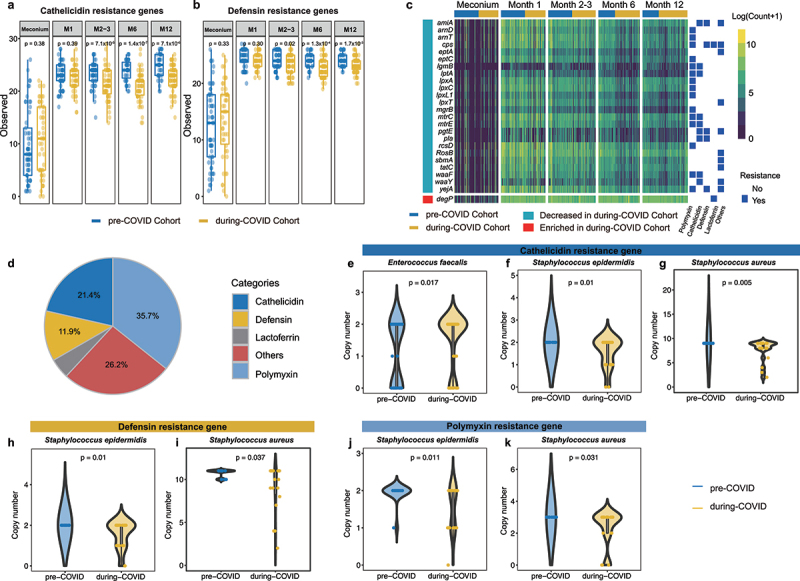
Distinct antimicrobial peptides (AMPs) resistance genes reservoir in infants during the COVID-19 pandemic (a) Data represents observed index of cathelicidin resistance genes in fecal sample. (b) Data represents observed index of defensin resistance genes in faecal sample. *p* values were given by Wilcoxon’s rank-sum tests. (c) The heatmap represents the abundance of differential AMP resistance genes in each sample quantified as logarithm of the hit counts [log (count +1)] for each AMP resistance gene. Differential AMP resistance genes were determined by a linear mixed-effects model with adjustments for chronological age, delivery mode, intrapartum antibiotics usage, and household furry pets using MaAsLin (all adjusted *p* < 0.05). (d) The pie chart represents the frequency of resistance class for the AMP resistance genes significantly affected by COVID-19 pandemic. The violin plot depicts the copy number of cathelicidin resistance genes in (e) *Enterococcus faecalis*, (f) *Staphylococcus epidermidis* and (g) *Staphylococcus aureus*; the copy number of defensin resistance genes in (h) *Staphylococcus epidermidis* and (i) *Staphylococcus aureus*, and the copy number of polymyxin resistance genes in (j) *Staphylococcus epidermidis* and (k) *Staphylococcus aureus* in pre-COVID and during-COVID cohort.

To address whether COVID-19 lockdown measures affect the richness of early life anti-microbial peptides (AMP), an evolutionarily conserved component of immune defense^26^ and a critical factor in early life gut microbiota seeding,^27^ shotgun metagenomic reads were mapped to a comprehensive dataset of 138 AMP resistance genes.^28^ We observed a significant decrease in the richness of AMP resistance genes in the stool of infants aged 2 months to 12 months born during the pandemic (Supplementary Figure S8A, Chao1: *P*_M2–3_ = 5 × 10^−5^; *P*_M6_ = 9.5 × 10^−8^; *P*_M12_ = 3.7 × 10^−4^; Supplementary Figure S5B, Observed: *P*_M2–3_ = 1.1 × 10^−5^; *P*_M6_ = 5.4 × 10^−10^; *P*_M12_ = 8.9×10^−7^). The richness of genes that confer resistance to two major human AMPs (cathelicidin and defensin) showed significant reduction at the age of 2–3 months (Figure 6a: cathelicidin: *P*_Observed_ = 7.1×10^−5^; Figure 6b: defensin: *P*_Observed_ = 0.02), 6 months (Figure 6a: cathelicidin: *P*_Observed_ = 1.4 × 10^−7^; Figure 6b: defensin: *P*_Observed_ = 1.3 × 10^−4^) and 12 months (Figure 6a: cathelicidin: *P*_Observed_ = 7.1×10^−6^; Figure 6b: defensin: *P*_Observed_ = 1.7 × 10^−3^) during the pandemic. Decreases in the richness of genes that confer resistance to cathelicidin and defensin from 2 to 12 months during the pandemic were also observed in a sensitivity analysis that excluded the infants who were exposed to antibiotics (Supplementary Figure S8C,D). We next compared the changes in the observed index within different time periods in the first year to determine whether longitudinal patterns of AMP resistance genes acquisition differ between the two cohorts. The changes in observed index from postpartum month 2–3 to month 1 and from month 12 to month 6 were significantly lower in during-COVID cohort than those in pre-COVID cohort (Supplementary Figure S8E). Additionally, the Chao1 index of AMP resistance genes was significantly lower in the high stringency group at 2–3 months of age compared to the low stringency group (Supplementary Figure S9A,B). MaAsLin analysis revealed that 24 AMP resistance genes were significantly decreased while only 1 gene, *degP*, was enriched in the during-COVID cohort when compared with the pre-COVID cohort (Figure 6c, FDR < 0.05). Among the AMP categories to which these AMP resistance genes belong, polymyxin, cathelicidin, and defensin ranked in the top three (Figure 6d).

To identify species that contributed to the difference in the abundance of these AMPs resistance genes between the two cohorts, we assembled and binned the high-quality paired-end reads into metagenome-assembled genomes (MAGs). Medium- and high-quality MAGs were annotated with AMP resistance genes. AMP resistance genes were detected in 547 out of 13,326 MAGs. After filtering away those species with a prevalence lower than 10%, we got 439 MAGs belonging to 8 species, including *Enterococcus faecalis*, *Klebsiella pneumoniae*, *Escherichia coli*, *Staphylococcus epidermidis*, *Staphylococcus aureus*, *Staphylococcus hominis*, *Intestinibacter bartlettii*, and *Klebsiella variicola*. Of these 8 species, only *Enterococcus faecalis* displayed a higher copy number of genes encoding for resistance to cathelicidin in during-COVID cohort compared with pre-COVID cohort (Figure 6e). By contrast, *Staphylococcus epidermidis* and *Staphylococcus aureus* harbored significantly lower genes that confer resistance to cathelicidin, defensin, and polymyxin in during-COVID cohort (Figure 6f–k). A sensitivity analysis that excluded the infants who were exposed to antibiotics showed consistent results (Supplementary Table S7).

### Infants born during COVID-19 pandemic had a distinct reservoir of gut antimicrobial resistance genes

**Figure 7.**
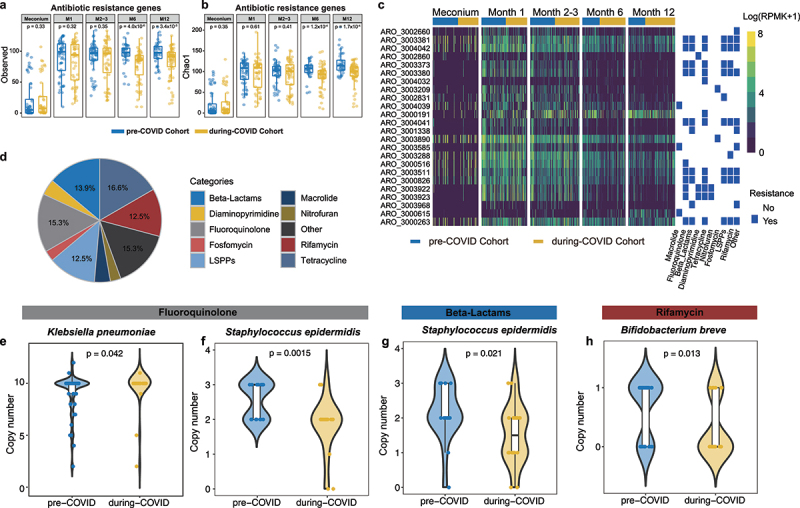
Distinct antibiotic resistance genes (ARGs) reservoir in infants during the COVID-19 pandemic (a) data represents observed index of ARGs in fecal samples. (b) Data represents Chao1 index of ARGs in faecal sample. *p* values were given by Wilcoxon’s rank-sum tests. (c) The heatmap represents ARGs abundance in each sample quantified as logarithm of the reads per kilobase per million [log (RPKM +1)] mapped reads for each ARG. Differential ARGs were determined by a linear mixed-effects model with adjustments for chronological age, delivery mode, intrapartum antibiotics usage, and household furry pets using MaAsLin (all adjusted *p* < 0.05). (d) The pie chart represents the frequency of resistance classes for the ARGs significantly affected by COVID-19 pandemic. The violin plot depicts the copy number of fluoroquinolone antibiotics resistance genes in (e) *Klebsiella pneumoniae* and (f) *Staphylococcus epidermidis*; the copy number of beta-lactams antibiotics resistance genes in (g) *Staphylococcus epidermidis*, and the copy number of rifamycin antibiotics resistance genes in (h) *Bifidobacterium breve* in pre-COVID and during-COVID cohort.

To characterize the impact of the COVID-19 pandemic measures on gut microbiota antibiotic resistance genes (ARGs) reservoir in infants’ gut microbiome, we performed ShortBRED to identify antibiotic resistance genes based on The Comprehensive Antibiotic Resistance Database (CARD).^29^ The richness indexes of ARGs were significantly lower in the gut microbiota of infants at 6 and 12 months old who were born during COVID-19 (Figure 7a,b, Observed index: *P*_M6_ = 4.0 × 10^−6^; *P*_M12_ = 3.4 × 10^−3^; Chao1 index: *P*_M6_ = 1.2 × 10^−4^; *P*_M12_ = 1.7 × 10^−4^). A sensitivity analysis that excludes the infants who were exposed to antibiotics showed consistent results of the difference in the richness of ARGs between the two cohorts. (Supplementary Figure S10A,B). Additionally, the observed index of ARGs resistance genes was significantly lower in the high stringency group at 12 months of age compared to the low stringency group (Supplementary Figure S10C,D).

A total of 24 reduced ARGs but no enriched ARG were found in the during-COVID cohort via longitudinal analysis (Figure 7c). These decreased ARGs encode resistance to major broad-spectrum antibiotic classes, with the highest representation of resistance to tetracycline, β-lactams, fluoroquinolones, and rifamycin (Figure 7d). Next, we investigated species that contributed to the difference in the abundance of these ARGs between the two cohorts by recovering medium- and high-quality MAGs, for which ARGs were assigned to 1240 MAGs. After filtering those species with a prevalence lower than 10%, we got 725 MAGs belonging to 12 species, including *Bifidobacterium bifidum*, *Bifidobacterium breve*, *Bifidobacterium dentium*, *Bifidobacterium longum*, *Bifidobacterium pseudocatenulatum*, *Enterococcus faecalis*, *Staphylococcus aureus*, *Staphylococcus epidermidis*, *Bacteroides fragilis*, *Bacteroides uniformis*, *Escherichia coli*, and *Klebsiella pneumoniae*. Notably, *Klebsiella pneumoniae* displayed a higher copy number of genes encoding for resistance to fluoroquinolone antibiotics in during-COVID cohort compared with pre-COVID though no specific ARG was found to be enriched in the during-COVID cohort (Figure 7e). Consistent with the reads-alignment analysis showing depression in β-lactams, fluoroquinolones, and rifamycin resistance genes, *Staphylococcus epidermidis* revealed decreased copy numbers of β-lactams and fluoroquinolones resistance genes, while *Bifidobacterium breve* harbored a lower copy number of rifamycin resistance genes in during-COVID group (Figure 7f–h). No specific species was found to be responsible for the difference in the abundance of tetracycline antibiotic resistance genes between the two cohorts. A sensitivity analysis that excluded the infants who were exposed to antibiotics showed consistent results (Supplementary Table S7).

## Discussion

This is the first proof-of-concept study to demonstrate the altered neonatal gut microbiome development during COVID-19 lockdown measures in an East Asian population. The data provide evidence that restrictive pandemic measures (including increased hygiene and sanitation measures) may have impaired neonatal microbial diversity. Using shotgun metagenomic sequencing of stool samples, we showed that the loss of microbiome diversity was associated with differences in microbial encoded function, including the reduction of bacteria richness and diversity related to antimicrobial peptides resistance genes and antibiotics resistance genes. Neonatal gut microbiome developmental trajectory during the COVID-19 pandemic was also changed when compared with infants born before the pandemic. These results in combination suggest a negative impact of the COVID-19 lockdown measures in relation to altered human gut microbiome development which may be associated with long-term health outcomes.

The first 1,000 days of life is a critical window for the establishment and development of the gut microbiota.^30,31^ In this early period, the immune system is in a state of immaturity, and an impaired immune response allows for gut microbiota colonization.^32,33^ During the COVID-19 pandemic, lockdown measures may affect the infant gut microbiome through: 1) Impairment of the maternal microbiome and 2) Reduced exposure to the environmental microbiome. The maternal microbiome serves as the major source of inoculation for the fetal gut, and is a critical factor in the development of the infant microbiome.^34^ The maternal microbiome is theorized to be affected during the COVID-19 pandemic due to poor microbial exposure, such as through reduced travel and decreased outings.^1^ Maternal-fetal transmission of gut microbes may also be impaired due to heightened hygiene practices, such as due to decreased frequency of breastfeeding and maternal self-isolation from the baby for fear of COVID-19 infection.^2^ In our study, COVID-19 pandemic lockdown measures were regarded as the major determinant impacting the maternal gut microbiota composition. Nevertheless, current evidence suggests that other factors other than mother-infant transmission may account for the differences in infant microbiome between our cohorts. To better understand how hygiene measures influence the relationship between maternal and infant microbiota, further studies with larger sample sizes are warranted. Indeed, infants may also have decreased exposure to the environmental microbiome through vigorous hygiene practices and decreased social exposure. A survey study in Hong Kong has previously shown that by March 2020, Hong Kong citizens had a high prevalence of practicing hygiene protective measures. The study reported that 93.0% of respondents admitted to washing hands more often, including with disinfectants or antiseptics, 89.6% practiced house disinfection, and 98.8% used face masks.^22^ The same study also showed that 85.1% of respondents avoided going to crowded places, and 83.8% stayed at home as much as possible. During COVID-19 in Hong Kong, policies such as the closure of schools and nursery day-care centers, and maximum gathering restrictions were implemented which further decreased exposure to community microbiome in the form of other individuals, such as other family members or peers.^35^ Such an increase in hygiene measures and self-isolation measures may have impaired the environmental microbiome colonization in the infant’s gut.

Altered infant gut microbial diversity is postulated to mediate the effects of environmental exposure and stress on later human health and diseases.^36^ Our results showed significant downregulation of the species *B. breve* and *B. dentium* at 2–3 months in the during-COVID cohort. *Bifidobacteria* have previously been shown to confer health benefits such as inducing host innate immunity.^37^ Depletion of *Bifidobacterial* species in early life has also been linked with immunological diseases.^38^ In addition, *Bifidobacteria* are generally considered to synthesize several B group vitamins and degrade hexose sugars through a particular metabolic pathway, termed the “bifid shunt”.^39^ Therefore, fewer *Bifidobacteria* species in the infant microbiome during the COVID-19 lockdown measures might be the reason for relevant deficient microbial functional pathways. Further work is warranted to elucidate the potential mechanism of whether microbiota modulation by *Bifidobacteria* species may serve as a potential approach to mitigate and restore gut microbiota dysbiosis. In contrast, no specific increased bacteria were identified to be associated with the increased energy generation pathways in the during-COVID cohort, indicating that augmentation observed in these pathways might be a cumulative effect of alterations in a series of bacterial populations in the during-COVID cohort. A potential underlying mechanism for the enhancement in energy generation pathways could be certain bacteria increase their reliance through upregulating energy metabolism under environmental stress, such as frequent usage of disinfection reagents during the COVID-19 pandemic. For example, a noticeable upregulation of genes and enzymes involved in energy metabolism, including the tricarboxylic acid (TCA) cycle, was identified in *Klebsiella pneumoniae* in response to antibiotic stress.^40,41^

We found that infants in the during-COVID cohort had reduced abundances of *Klebsiella pneumoniae*, *Klebsiella quasipneumoniae*, and *Klebsiella variicola* at months 6 and 12 compared with that in the pre-COVID cohort, which may be related to the stricter epidemic control, the isolation of children at home, the reduction of hospital visits, the avoidance of crowd gathering and the widespread use of disinfectants. Similarly, the prevalence rate of *Klebsiella pneumoniae* infection in pediatric patients during the COVID-19 pandemic was reported to be decreased.^42,43^

Infant gut microbial diversity loss may also be due to poorer microbiome adaptability. The immune response to exogenous stimuli is tightly regulated during early life. Newborns have an immature cellular defense immune system and are more susceptible to infections. Antimicrobial peptides therefore provide a compensatory innate defense mechanism during the development of cellular immune response mechanisms in the newborn period and induce highly specific changes in the composition of the human microbiota with profound implications for disease risks.^28,44^ We applied a comprehensive approach to systematically characterize the potential impact of pandemic measures on the richness of AMP and antibiotic resistance gene reservoirs in infant’s gut microbiome. Our results showed that the richness of AMPs and/or antibiotics resistance genes was indeed compromised in neonates during the COVID-19 pandemic, suggesting compromised gut microbiome adaptability. On the other hand, the decrease in richness of ARGs in during-COVID cohort may be related to the reduction of hospital visits and decreased antibiotics consumption during the pandemic.^45,46^

We furthermore used metagenome-assembled genomes to compare the microbial origin of AMP and antibiotics resistance genes at the strain level. Notably, a skin commensal, *Staphylococcus epidermidis*, and a common skin pathogen, *Staphylococcus aureus* were found to harbor a lower copy number of cathelicidin, defensin, and polymyxin resistance genes in during-COVID cohort. In addition, *Staphylococcus epidermidis* also carried a lower copy number of fluoroquinolone and beta-lactams antibiotics resistance genes in the during-COVID cohort. This may be due to the reduction in community exposure of infants, the widespread use of disinfectants, and increased frequency of handwashing of their caregivers. Nevertheless, we still observed that *Klebsiella pneumoniae* possessed a higher copy number of fluoroquinolone antibiotic resistance genes in infants born during the COVID-19 pandemic, which alerts us to the growing threat of multi-drug resistant *Klebsiella pneumoniae* infection in the post-COVID-19 era. This is consistent with a previous study showing that *Klebsiella* genus possessed evident correlations with multiple ARGs in the hospital wastewater samples during the COVID-19 pandemic,^47^ which may be a source for the dissemination of multi-drug resistant *Klebsiella* in the environment.^48^

Such changes to the infant gut microbiome may affect the development of the immune system, and predispose to the development of atopic, metabolic, and inflammatory diseases.^49^ However, more research is needed to delineate the potential mechanisms. Longitudinal follow-up of infants in these two cohorts to compare the growth status and incidence of diseases is necessary to investigate the long-term effect on the health of the changes in gut microbiota in early life due to the COVID-19 pandemic.

The main strength of this proof-of-concept study is the replication and extension design of the during-COVID cohort compared with the pre-COVID cohort with regular longitudinal clinical follow-up at the same clinical research site. Collecting fecal samples longitudinally from birth, 1 month, 2-3 months, 6 months, and until 12 months of age allowed for the characterization of the microbial population during the first year of life. Especially, the reasonably large cohorts’ sizes recruited and maintained during the pandemic. Another major strength is the use of shotgun metagenomics. Previous studies on the gut microbiome relied on 16S rRNA gene sequencing. The use of shotgun metagenomics in this study allows for better characterization of the gut microbiome in infants, with identification of low-abundant taxa.^50^ Shotgun metagenomics also allows for increased resolution and better functional classification of the gut microbiome, such as pertaining to that of AMPs and ARGs.^51^

Our study has some limitations. The sample size is modest, and external validation cohorts of different ethnogeography populations are needed. There is no evidence directly connecting hygiene practices to our findings since it is difficult to ascertain individual lifestyle responses (such as the exact solid food introduced time), social isolation status of infants, and behavior toward pandemic control measures. However, standardized measures in maternity wards are applied across all hospitals and outpatient settings in Hong Kong. Although gender, age, education, household income, and occupation can affect adherence to pandemic controls, most of the public in Hong Kong during the early pandemic were compliant.^52,53^ Lastly, while our study has shown microbial compositional changes and possible functional alterations in the during-COVID cohort compared with pre-COVID infants, more research is needed to fully characterize whether this represents a pathological state, and what constitutes a healthy microbiome. This study makes the assumption that a pre-COVID state may represent a less pathological state based on the hygiene hypothesis. Future research can focus on elucidating the exact biological pathways that modulate microbiome-immune system cross-talk and development. This highlights the need for large-scale, long-term cohort follow-up studies to better understand the consequences of the COVID-19 pandemic and establish whether the alteration of the neonatal microbiota during the pandemic would negatively affect health outcomes in childhood and later life.

## Materials and methods

### Cohort description and study subjects

We included two longitudinal cohorts from Hong Kong during (1) Oct 2017-Jan 2020, defined as the pre-COVID cohort; and (2) April 2020-Jan 2022, defined as the during-COVID cohort. Both cohorts recruited neonates who were delivered at the Prince of Wales Hospital in Hong Kong. 521 stool samples were collected at birth, 1 month, 2-3 months, 6 months, and until 12 months postpartum from 67 pre-COVID and 67 matched during-COVID infants. 103 stool samples were collected from mothers in the 2^nd^ to 3^rd^ trimester, including 44 samples in the pre-COVID group and 59 samples in the during-COVID group.

### Faecal samples

Faecal samples were collected at home by all subjects using tubes prepared by investigators containing preservative media (cat. 28330, Norgen Biotek Corp, Ontario Canada). The Norgen preservative can preserve and allow safe transportation of microbial DNA & RNA at ambient temperature eliminating sample variability. The stool sample was sent to the hospital within 24 h of collection and stored at − 80°C freezers until further processing.

### Metagenomic sequencing of faecal samples

The fecal DNA was extracted using the DNeasy PowerSoil Pro Kit (QIAGEN, Hilden, Germany) following the manufacturer’s instructions. Libraries were prepared from the extracted DNA using the Illumina DNA Prep (Illumina, California USA), and sequenced with paired-end 150 bp sequencing strategy by Illumina NovaSeq 6000 System at the Microbiota I-Center (MagIC), Hong Kong Science Park. Microbial Community Standard (ZymoBIOMICS™) was subject to repeated extraction, library construction and metagenome sequencing to assess batch variation. Norgen preservative media was used as negative controls during DNA extraction, library construction, and sequencing. Raw sequence reads were filtered and quality-trimmed using Trimmomatic (v0.39).^54^ The mean read length and number of sequences provided on the MultiQC reports were used to determine sequencing coverage. Human-derived reads were filtered using Kneaddata (v0.10.0) based on a human reference genome (hg19) with default parameters. Profiling of bacterial communities was performed using MetaPhlAn3 (v3.0.14)^55^ by mapping reads to clade-specific markers. Microbial alpha diversity indices were calculated based on the species-level profiles, including Chao1 index and observed species, Shannon diversity and phylogenetic diversity. Beta diversity was assessed with Bray-Curtis dissimilarity using R packages phyloseq and vegan, and was visualized by principal component analysis (PCA). The sample collection, storage, and sequencing strategies of both cohorts were consistent.

### Strain-level profiling

Strain-level analysis was performed using a combination of gene-content-based profiling using PanPhlAn,^56^ and single-nucleotide variant profiling using StrainPhlAn^57^ with default parameters. Strain distance was defined as for PanPhlAn including normalization of each tree by its median value. A pair of strains with a strain distance lower than 0.1 was considered the same strain.^58^ Strain-sharing rates were calculated as the number of shared strains divided by the number of species common to each pair of individuals.^59^

### Gut microbiota maturation estimation

For estimated gut microbial age, Random Forest regression was applied to regress with the relative abundances of bacterial species (prevalence >5%) of a training set made of infants in the pre-COVID cohort after filtering out all species with a prevalence less than 5% against their chronologic age using default parameters of the R implementation of the algorithm (R package randomForest, ntree = 10,000, default mtry). Ranked lists of species in order of feature importance based on the percent increase in mean squared error reported by Random Forest were determined over 20 iterations of the algorithm. Higher values of the percent increase in mean square error indicate more significant variables. To estimate the minimal number of top-ranking age-discriminatory species required for prediction, the rfcv function implemented in the randomForest package was applied with 10-fold cross-validations over 20 times. The minimal number of age-discriminatory species was determined by minimizing the cross-validation error. A sparse model consisting of the top 33 taxa was then trained on the training set followed by internal validation using bootstrapping. Random Forest model performance was evaluated using mean absolute error (MAE) and Kendall’s tau coefficient by correlating estimated gut microbiota age and chronological age at sampling. The relative microbiota maturity was defined as the deviation from a smooth spline fit of microbiota age values with respect to the chronologic age of healthy infants in the pre-COVID cohort.

### Profiling of microbial functional pathways

Bacterial functions were predicted using HUMANN3.0.1. Data on the functionality was normalized based on relative log expression by Deseq2 (v1.26.0).^60^ To determine differential pathways between the pre-COVID cohort and the during-COVID cohort during the first year, we applied Deseq2 to identify differential pathways at each time period, including 1 month, 2–3 months, 6 months, and 12 months followed by MaAsLin^61^ to adjust delivery mode, intrapartum antibiotics usage, chronological age, and household furry pets.

### Profiling of antimicrobial peptide resistance genes and antibiotic resistance genes

To detect AMP resistance genes, we performed a sequence similarity search against a manually curated collection of AMP resistance genes using Diamond (BLASTx mode).^62^ The raw sequencing data in FASTQ format was first converted to FASTA format before aligning with the AMP.dmnd file. After the alignment, we processed the output to obtain hit counts for each AMP resistance gene. The counts of resistance genes were then normalized using the rlog transformation implemented in DESeq2.^60^ To identify antibiotic resistance genes, we employed ShortBRED,^29^ which is based on the Comprehensive Antibiotic Resistance Database (CARD).^29^ Differential AMP resistance genes and ARGs between the two cohorts were determined by MaAsLin to adjust delivery mode, intrapartum antibiotics usage, chronological age, and household furry pets.

### Analysis of AMP resistance genes and ARGs on metagenome-assembled genomes

High-quality paired-end reads were assembled and binned into metagenome-assembled genomes (MAGs) using the Metapi pipeline with associated dependencies.^63^ Average nucleotide identity (ANI) from MAGs was calculated by skani v.0.1.5.^64^ Then, MAGs were clustered into strain-level genomes using python networkX package based on the 0.99 ANI cutoff.^65^ Representative strain MAGs were generated for downstream analysis. Taxonomic classification was assigned using GTDBTK v2.3.2 based on GTDB Release212 database.^66^ Taxonomy dump information was generated using taxonkit v0.15.0.^67^ Medium- and high-quality strain-level MAGs were aligned with the AMP.dmnd file using diamond V2.1.8 for AMP annotation and ARGs were annotated by ABRicate v1.0.1.^68,69^ The results were filtered at > 95% query coverage (read coverage) and > 80% alignment identity thresholds. Hit counts for AMP resistance genes and ARGs of each MAGs were obtained. To identify species that contributed to the difference in the abundance of resistance genes for certain AMP classes and antibiotics classes between the two cohorts, the hit counts for genes that were resistant to the specific AMP class or antibiotics class in a MAG were summed. MAGs that belong to those species with a prevalence lower than 10% were removed.

## Quantification and statistical analysis

Propensity matching was used to adjust sex, delivery mode, and breastfeeding practice between the pre-COVID cohort and during-COVID cohort. One-to-one propensity matching was performed with the MatchIt package in R using the nearest neighbor approach and the standardized mean difference of all adjusted factors between the two groups was not more than 0.1.^70^ Categorical variables were presented as counts (percentage). Changes in continuous variables, including the relative abundances of bacteria were compared by Wilcoxon rank-sum test, whereas changes in categorical variables were compared using the Chi-square test or Fisher’s exact test. A two-sided p-value of <5% was considered statistically significant. The development of alpha diversity along chronological age was measured by Kendall rank correlation coefficient and the difference in development rates between the two groups was compared by Fisher’s Z Transformation after converting Kendall’s Tau to Pearson’s r. Pairwise multi-level comparisons in each timepoint of the pre-COVID cohort and the during-COVID cohort were carried out on the Bray-Curtis dissimilarity matrix using pairwise Adonis test assessed using permutational multivariate analysis of variance (PERMANOVA). Differential species, pathways, ARGs, and AMP resistance genes between the two groups within the first year were detected using Multivariate Analysis by Linear Models (MaAsLin), including cohorts, delivery mode, intrapartum antibiotics usage, household furry pets, and chronological age as fixed effects and subjects as random effect. Differentially abundant species at each sample collection timepoint between the two groups were identified using the linear discriminant analysis (LDA) effect size (LEfSe) implemented in the Huttenhower Lab Galaxy Server (http://huttenhower.sph.harvard.edu/galaxy/.) with cutoffs being LDA score >2, and *p* < 0.05. Correlations between bacteria species and functional pathways were assessed with Hierarchical All-against-All association testing (HAIIA).^71^ All microbiome-related statistical tests were performed with R Statistics (version 4.0.3). For a sensitivity analysis, we excluded infants who were exposed to antibiotics and investigated the differences in gut microbiota alpha diversity, composition, gut microbial relative maturity, and richness of gut microbial AMP and ARGs resistance genes, as well as differences in the copy number of AMP and ARGs resistance genes in specific bacteria between pre-COVID and during-COVID cohorts.

## Supplementary Material

Supplemental Material

## Data Availability

This study did not generate new unique codes. Quality-controlled and human DNA-removed sequence data generated for this study are available in the Sequence Read Archive database under BioProject accession PRJNA1031773. (https://www.ncbi.nlm.nih.gov/bioproject/PRJNA1031773.)

## References

[1] Finlay BB, Amato KR, Azad M, Blaser MJ, Bosch TCG, Chu H, Dominguez-Bello MG, Ehrlich SD, Elinav E, Geva-Zatorsky N, Gros P, et al. The hygiene hypothesis, the COVID pandemic, and consequences for the human microbiome. Proc Natl Acad Sci. 2021;118(6):e2010217118. doi:10.1073/pnas.2010217118. 33472859 PMC8017729

[2] Romano-Keeler J, Zhang J, Sun J. COVID-19 and the neonatal microbiome: will the pandemic cost infants their microbes? Gut Microbes. 2021;13(1):1912562. doi:10.1080/19490976.2021.1912562. 33960272 PMC8115548

[3] Johnson CC, Ownby DR. Allergies and asthma: do atopic disorders result from inadequate immune homeostasis arising from infant gut dysbiosis? Expert Rev Clin Immunol. 2016;12(4):379–21. doi:10.1586/1744666X.2016.1139452. 26776722 PMC4829075

[4] Asher MI, Montefort S, Björkstén B, Lai CK, Strachan DP, Weiland SK, Williams H. Worldwide time trends in the prevalence of symptoms of asthma, allergic rhinoconjunctivitis, and eczema in childhood: ISAAC phases one and three repeat multicountry cross-sectional surveys. Lancet. 2006;368(9537):733–743. doi:10.1016/S0140-6736(06)69283-0. 16935684

[5] Asher MI, Rutter CE, Bissell K, Chiang C-Y, El Sony A, Ellwood E, Ellwood P, García-Marcos L, Marks GB, Morales E, et al. Worldwide trends in the burden of asthma symptoms in school-aged children: global asthma network phase I cross-sectional study. Lancet. 2021;398(10311):1569–1580. doi:10.1016/S0140-6736(21)01450-1. 34755626 PMC8573635

[6] Dharmage SC, Perret JL, Custovic A. Epidemiology of asthma in children and adults. Front Pediatr. 2019;7:246. doi:10.3389/fped.2019.00246. 31275909 PMC6591438

[7] Song P, Adeloye D, Salim H, Dos Santos JP, Campbell H, Sheikh A, Rudan I. Global, regional, and national prevalence of asthma in 2019: a systematic analysis and modelling study. J Glob Health. 2022;12:12. doi:10.7189/jogh.12.04052. PMC9239324 35765786

[8] Haahtela T, Holgate S, Pawankar R, Akdis CA, Benjaponpitak S, Caraballo L, Demain J, Portnoy J, von Hertzen L. The biodiversity hypothesis and allergic disease: world allergy organization position statement. World Allergy Organ J. 2013;6:3. doi:10.1186/1939-4551-6-3. 23663440 PMC3646540

[9] Rook GA. Hygiene hypothesis and autoimmune diseases. Clinic Rev Allerg Immunol. 2012;42(1):5–15. doi:10.1007/s12016-011-8285-8. 22090147

[10] Von Hertzen L, Hanski I, Haahtela T. Natural immunity: biodiversity loss and inflammatory diseases are two global megatrends that might be related. EMBO Rep. 2011;12(11):1089–1093. doi:10.1038/embor.2011.195. 21979814 PMC3207110

[11] Frei R, Lauener R, Crameri R, O’Mahony L. Microbiota and dietary interactions–an update to the hygiene hypothesis? Allergy. 2012;67(4):451–461. doi:10.1111/j.1398-9995.2011.02783.x. 22257145

[12] Rothschild D, Weissbrod O, Barkan E, Kurilshikov A, Korem T, Zeevi D, Costea PI, Godneva A, Kalka IN, Bar N, et al. Environment dominates over host genetics in shaping human gut microbiota. Nature. 2018;555(7695):210–215. doi:10.1038/nature25973. 29489753

[13] Adlerberth I, Wold A. Establishment of the gut microbiota in Western infants. Acta paediatrica. 2009;98(2):229–238. doi:10.1111/j.1651-2227.2008.01060.x. 19143664

[14] Lynch SV, Wood RA, Boushey H, Bacharier LB, Bloomberg GR, Kattan M, O’Connor GT, Sandel MT, Calatroni A, Matsui E, et al. Effects of early-life exposure to allergens and bacteria on recurrent wheeze and atopy in urban children. J Allergy Clin Immunol. 2014;134(3):593–601.e12. doi:10.1016/j.jaci.2014.04.018. 24908147 PMC4151305

[15] Sbihi H, Boutin RC, Cutler C, Suen M, Finlay BB, Turvey SE. Thinking bigger: how early‐life environmental exposures shape the gut microbiome and influence the development of asthma and allergic disease. Allergy. 2019;74(11):2103–2115. doi:10.1111/all.13812. 30964945

[16] Yang Z, Chen Z, Lin X, Yao S, Xian M, Ning X, Fu W, Jiang M, Li N, Xiao X, et al. Rural environment reduces allergic inflammation by modulating the gut microbiota. Gut Microbes. 2022;14(1):2125733. doi:10.1080/19490976.2022.2125733. 36193874 PMC9542937

[17] Theodorou J, Nowak E, Böck A, Salvermoser M, Beerweiler C, Zeber K, Kulig P, Tsang MS, Wong C-K, Wong GWK, et al. Mitogen-activated protein kinase signaling in childhood asthma development and environment-mediated protection. Pediatr Allergy Immunol. 2022;33(1):e13657. doi:10.1111/pai.13657. 34455626

[18] Gillman MW. Developmental origins of health and disease. N Engl J Med. 2005;353(17):1848. doi:10.1056/NEJMe058187. 16251542 PMC1488726

[19] McGuire M, McGuire MA, Bode L. Prebiotics and probiotics in human milk: origins and functions of milk-borne oligosaccharides and bacteria. Academic Press; 2016.

[20] Wong MCS, Ng RWY, Chong KC, Lai CKC, Huang J, Chen Z, Boon SS, Chan PKS. Stringent containment measures without complete city lockdown to achieve low incidence and mortality across two waves of COVID-19 in Hong Kong. BMJ Glob Health. 2020;5(10):e003573. doi:10.1136/bmjgh-2020-003573. PMC7542625 33028700

[21] Chan H-Y, Chen A, Ma W, Sze N-N, Liu X. COVID-19, community response, public policy, and travel patterns: a tale of Hong Kong. Transp Policy. 2021;106:173–184. doi:10.1016/j.tranpol.2021.04.002. PMC8026218 33846671

[22] Cowling BJ, Ali ST, Ng TW, Tsang TK, Li JCM, Fong MW, Liao Q, Kwan MY, Lee SL, Chiu SS, et al. Impact assessment of non-pharmaceutical interventions against coronavirus disease 2019 and influenza in Hong Kong: an observational study. Lancet Publ Health. 2020;5(5):e279–e288. doi:10.1016/S2468-2667(20)30090-6. PMC7164922 32311320

[23] Singh A. Covid-19: disinfectants and sanitisers are changing microbiomes. Bmj. 2020;370:m2795. doi:10.1136/bmj.m2795. 32665219

[24] Munyaka PM, Khafipour E, Ghia J-E. External influence of early childhood establishment of gut microbiota and subsequent health implications. Front Pediatr. 2014;2:109. doi:10.3389/fped.2014.00109. 25346925 PMC4190989

[25] Subramanian S, Huq S, Yatsunenko T, Haque R, Mahfuz M, Alam MA, Benezra A, DeStefano J, Meier MF, Muegge BD, et al. Persistent gut microbiota immaturity in malnourished Bangladeshi children. Nature. 2014;510(7505):417–421. doi:10.1038/nature13421. 24896187 PMC4189846

[26] Hancock RE, Haney EF, Gill EE. The immunology of host defence peptides: beyond antimicrobial activity. Nat Rev Immunol. 2016;16(5):321–334. doi:10.1038/nri.2016.29. 27087664

[27] Xiao L, Zhao F. Microbial transmission, colonisation and succession: from pregnancy to infancy. Gut. 2023;72(4):772–786. doi:10.1136/gutjnl-2022-328970. 36720630 PMC10086306

[28] Kintses B, Méhi O, Ari E, Számel M, Györkei Á, Jangir PK, Nagy I, Pál F, Fekete G, Tengölics R, et al. Phylogenetic barriers to horizontal transfer of antimicrobial peptide resistance genes in the human gut microbiota. Nat Microbiol. 2018;4(3):447–458. doi:10.1038/s41564-018-0313-5. 30559406 PMC6387620

[29] Kaminski J, Gibson MK, Franzosa EA, Segata N, Dantas G, Huttenhower C. High-specificity targeted functional profiling in microbial communities with ShortBRED. PLOS Comput Biol. 2015;11(12):e1004557. doi:10.1371/journal.pcbi.1004557. 26682918 PMC4684307

[30] Zeng S, Ying J, Li S, Qu Y, Mu D, Wang S. First 1000 days and beyond after birth: gut microbiota and necrotizing enterocolitis in preterm infants. Front Microbiol. 2022;13:905380. doi:10.3389/fmicb.2022.905380. 35801107 PMC9253634

[31] Robertson RC, Manges AR, Finlay BB, Prendergast AJ. The human microbiome and child growth – first 1000 days and beyond. Trends in Microbiol. 2019;27(2):131–147. doi:10.1016/j.tim.2018.09.008. 30529020

[32] Zheng D, Liwinski T, Elinav E. Interaction between microbiota and immunity in health and disease. Cell Res. 2020;30(6):492–506. doi:10.1038/s41422-020-0332-7. 32433595 PMC7264227

[33] Belkaid Y, Hand TW. Role of the microbiota in immunity and inflammation. Cell. 2014;157(1):121–141. doi:10.1016/j.cell.2014.03.011. 24679531 PMC4056765

[34] Mueller NT, Bakacs E, Combellick J, Grigoryan Z, Dominguez-Bello MG. The infant microbiome development: mom matters. Trends Mol Med. 2015;21(2):109–117. doi:10.1016/j.molmed.2014.12.002. 25578246 PMC4464665

[35] Viner RM, Russell SJ, Croker H, Packer J, Ward J, Stansfield C, Mytton O, Bonell C, Booy R. School closure and management practices during coronavirus outbreaks including COVID-19: a rapid systematic review. Lancet Child & Adolesc Health. 2020;4(5):397–404. doi:10.1016/S2352-4642(20)30095-X. PMC7270629 32272089

[36] Naspolini NF, Meyer A, Moreira JC, Sun H, Froes-Asmus CIR, Dominguez-Bello MG. Environmental pollutant exposure associated with altered early-life gut microbiome: results from a birth cohort study. Environ Res. 2022;205:112545. doi:10.1016/j.envres.2021.112545. 34896087

[37] Konieczna P, Akdis CA, Quigley EMM, Shanahan F, O’Mahony L. Portrait of an immunoregulatory bifidobacterium. Gut Microbes. 2012;3(3):261–266. doi:10.4161/gmic.20358. 22572827 PMC3427218

[38] O’Neill I, Schofield Z, Hall LJ, Marchesi JR. Exploring the role of the microbiota member bifidobacterium in modulating immune-linked diseases. Emerging Top Life Sci. 2017;1(4):333–349. doi:10.1042/ETLS20170058. PMC7288987 33525778

[39] Derrien M, Turroni F, Ventura M, van Sinderen D. Insights into endogenous bifidobacterium species in the human gut microbiota during adulthood. Trends Microbiol. 2022;30(10):940–947. doi:10.1016/j.tim.2022.04.004. 35577716

[40] Khan A, Sharma D, Faheem M, Bisht D, Khan AU. Proteomic analysis of a carbapenem-resistant Klebsiella pneumoniae strain in response to meropenem stress. J Global Antimicrob Resist. 2017;8:172–178. doi:10.1016/j.jgar.2016.12.010. 28219823

[41] Van Laar TA, Chen T, You T, Leung KP. Sublethal concentrations of carbapenems alter cell morphology and genomic expression of Klebsiella pneumoniae biofilms. Antimicrob Agents Chemother. 2015;59(3):1707–1717. doi:10.1128/AAC.04581-14. 25583711 PMC4325768

[42] Du B, Sun M, Qin X, Wang H, Sun J, Li J, Zhang X, Zhang W. The influences of the COVID-19 pandemic on Klebsiella pneumoniae infection in children, Henan, China, 2018–2022. Indian J Microbiol. 2024;64(1):264–266. doi:10.1007/s12088-023-01177-3. 38468729 PMC10924817

[43] Patil S, Chen H, Dong S, Liu S, Wen F. Klebsiella pneumoniae infection in the paediatric population before and after the COVID-19 pandemic in Shenzhen, China. J Infect. 2023;86(3):256–308. doi:10.1016/j.jinf.2023.01.003. PMC9860501 36632941

[44] Dorschner RA, Lin KH, Murakami M, Gallo RL. Neonatal skin in mice and humans expresses increased levels of antimicrobial peptides: innate immunity during development of the adaptive response. Pediatr Res. 2003;53(4):566–572. doi:10.1203/01.PDR.0000057205.64451.B7. 12612195

[45] Fukushige M, Ngo NH, Lukmanto D, Fukuda S, Ohneda O. Effect of the COVID-19 pandemic on antibiotic consumption: a systematic review comparing 2019 and 2020 data. Front Public Health. 2022;10:946077. doi:10.3389/fpubh.2022.946077. 36330124 PMC9623150

[46] Wang T, Shen L, Yin J, Zhou L, Sun Q. Antibiotic use in township hospitals during the COVID-19 pandemic in Shandong, China. Antimicrob Resist Infect Control. 2022;11(1):164. doi:10.1186/s13756-022-01206-8. 36566210 PMC9789504

[47] Zhao L, Lv Z, Lin L, Li X, Xu J, Huang S, Chen Y, Fu Y, Peng C, Cao T, et al. Impact of COVID-19 pandemic on profiles of antibiotic-resistant genes and bacteria in hospital wastewater. Environ Pollut. 2023;334:122133. doi:10.1016/j.envpol.2023.122133. 37399936

[48] Ndlovu T, Kgosietsile L, Motshwarakgole P, Ndlovu SI. Evaluation of potential factors influencing the dissemination of multidrug-resistant Klebsiella pneumoniae and alternative treatment strategies. TropicalMed. 2023;8(8):381. doi:10.3390/tropicalmed8080381. PMC10459473 37624319

[49] Wong E, Lui K, Day AS, Leach ST. Manipulating the neonatal gut microbiome: current understanding and future perspectives. Arch Dis Child Fetal Neonatal Ed. 2022;107(4):346–350. doi:10.1136/archdischild-2021-321922. 34433586 PMC9209688

[50] Durazzi F, Sala C, Castellani G, Manfreda G, Remondini D, De Cesare A. Comparison between 16S rRNA and shotgun sequencing data for the taxonomic characterization of the gut microbiota. Sci Rep. 2021;11(1):3030. doi:10.1038/s41598-021-82726-y. 33542369 PMC7862389

[51] Jovel J, Patterson J, Wang W, Hotte N, O’Keefe S, Mitchel T, Perry T, Kao D, Mason AL, Madsen KL, et al. Characterization of the gut microbiome using 16S or shotgun metagenomics. Front Microbiol. 2016;7:459. doi:10.3389/fmicb.2016.00459. 27148170 PMC4837688

[52] Chan EYY, Kim JH, Kwok K-O, Huang Z, Hung KKC, Wong ELY, Lee EKP, Wong SYS. Population adherence to infection control behaviors during Hong Kong’s first and third COVID-19 waves: a serial cross-sectional study. Int J Environ Res Pub Health. 2021;18(21):11176. doi:10.3390/ijerph182111176. 34769694 PMC8583559

[53] Reicher S, Drury J. Pandemic fatigue? How adherence to COVID-19 regulations has been misrepresented and why it matters. Bmj. 2021;372:n137. doi:10.1136/bmj.n137. 33461963

[54] Bolger AM, Lohse M, Usadel B. Trimmomatic: a flexible trimmer for Illumina sequence data. Bioinformat. 2014;30(15):2114–2120. doi:10.1093/bioinformatics/btu170. PMC4103590 24695404

[55] Beghini F, McIver LJ, Blanco-Míguez A, Dubois L, Asnicar F, Maharjan S, Mailyan A, Manghi P, Scholz M, Thomas AM, et al. Integrating taxonomic, functional, and strain-level profiling of diverse microbial communities with bioBakery 3. Elife. 2021;10:10. doi:10.7554/eLife.65088. PMC8096432 33944776

[56] Scholz M, Ward DV, Pasolli E, Tolio T, Zolfo M, Asnicar F, Truong DT, Tett A, Morrow AL, Segata N. Strain-level microbial epidemiology and population genomics from shotgun metagenomics. Nat Methods. 2016;13(5):435–438. doi:10.1038/nmeth.3802. 26999001

[57] Truong DT, Tett A, Pasolli E, Huttenhower C, Segata N. Microbial strain-level population structure and genetic diversity from metagenomes. Genome Res. 2017;27(4):626–638. doi:10.1101/gr.216242.116. 28167665 PMC5378180

[58] Ferretti P, Pasolli E, Tett A, Asnicar F, Gorfer V, Fedi S, Armanini F, Truong DT, Manara S, Zolfo M, et al. Mother-to-infant microbial transmission from different body sites shapes the developing infant gut microbiome. Cell Host & Microbe. 2018;24(1):133–145.e5. doi:10.1016/j.chom.2018.06.005. 30001516 PMC6716579

[59] Dubois L, Valles-Colomer M, Ponsero A, Helve O, Andersson S, Kolho K-L, Asnicar F, Korpela K, Salonen A, Segata N, et al. Paternal and induced gut microbiota seeding complement mother-to-infant transmission. Cell Host & Microbe. 2024;32(6):1011–1024.e4. doi:10.1016/j.chom.2024.05.004. 38870892

[60] Love MI, Huber W, Anders S. Moderated estimation of fold change and dispersion for RNA-seq data with DESeq2. Genome Biol. 2014;15(12):550. doi:10.1186/s13059-014-0550-8. 25516281 PMC4302049

[61] Mallick H, Rahnavard A, McIver LJ, Ma S, Zhang Y, Nguyen LH, Tickle TL, Weingart G, Ren B, Schwager EH, et al. Multivariable association discovery in population-scale meta-omics studies. PLOS Comput Biol. 2021;17(11):e1009442. doi:10.1371/journal.pcbi.1009442. 34784344 PMC8714082

[62] Buchfink B, Reuter K, Drost HG. Sensitive protein alignments at tree-of-life scale using DIAMOND. Nat Methods. 2021;18(4):366–368. doi:10.1038/s41592-021-01101-x. 33828273 PMC8026399

[63] Zhu J, Tian L, Chen P, Han M, Song L, Tong X, Sun X, Yang F, Lin Z, Liu X, et al. Over 50,000 metagenomically assembled draft genomes for the human oral microbiome reveal new taxa. Genom, Proteom & Bioinf. 2022;20(2):246–259. doi:10.1016/j.gpb.2021.05.001. PMC9684161 34492339

[64] Shaw J, Yu YW. Fast and robust metagenomic sequence comparison through sparse chaining with skani. Nat Methods. 2023;20(11):1661–1665. doi:10.1038/s41592-023-02018-3. 37735570 PMC10630134

[65] Hagberg A, Swart P, Chult S. Exploring network structure, dynamics, and function using network. Los Alamos (NM) (United States): Los Alamos National Lab (LANL); 2008.

[66] Chaumeil PA, Mussig AJ, Hugenholtz P, Parks DH. GTDB-Tk v2: memory friendly classification with the genome taxonomy database. Bioinformat. 2022;38(23):5315–5316. doi:10.1093/bioinformatics/btac672. PMC9710552 36218463

[67] Shen W, Ren H. TaxonKit: a practical and efficient NCBI taxonomy toolkit. J Genet Genom. 2021;48(9):844–850. doi:10.1016/j.jgg.2021.03.006. 34001434

[68] GitHub TS.

[69] Jia B, Raphenya AR, Alcock B, Waglechner N, Guo P, Tsang KK, Lago BA, Dave BM, Pereira S, Sharma AN, et al. CARD 2017: expansion and model-centric curation of the comprehensive antibiotic resistance database. Nucleic Acids Res. 2017;45(D1):D566–d573. doi:10.1093/nar/gkw1004. 27789705 PMC5210516

[70] Ho D, Imai K, King G. Package ‘matchIt’. Version [Google Scholar]. 2018.

[71] Ghazi AR, Sucipto K, Rahnavard A, Franzosa EA, McIver LJ, Lloyd-Price J, Schwager E, Weingart G, Moon YS, Morgan XC, et al. High-sensitivity pattern discovery in large, paired multiomic datasets. Bioinformat. 2022;38(Supplement_1):i378–i385. doi:10.1093/bioinformatics/btac232. PMC9235493 35758795

