# Supplementary material for: Impacts of COVID-19 pandemic on early life gut microbiome
Source: Gut Microbes. 2024 Dec 31;17(1):2443117. doi: 10.1080/19490976.2024.2443117 (PMC12931694; doi:10.1080/19490976.2024.2443117)
Supplement: Supplemental Material [file KGMI_A_2443117_SM0176.zip › kgmi-s-2024-1468-20241212204651/suppl_data/Supplementary_Figure_clean.docx]

**
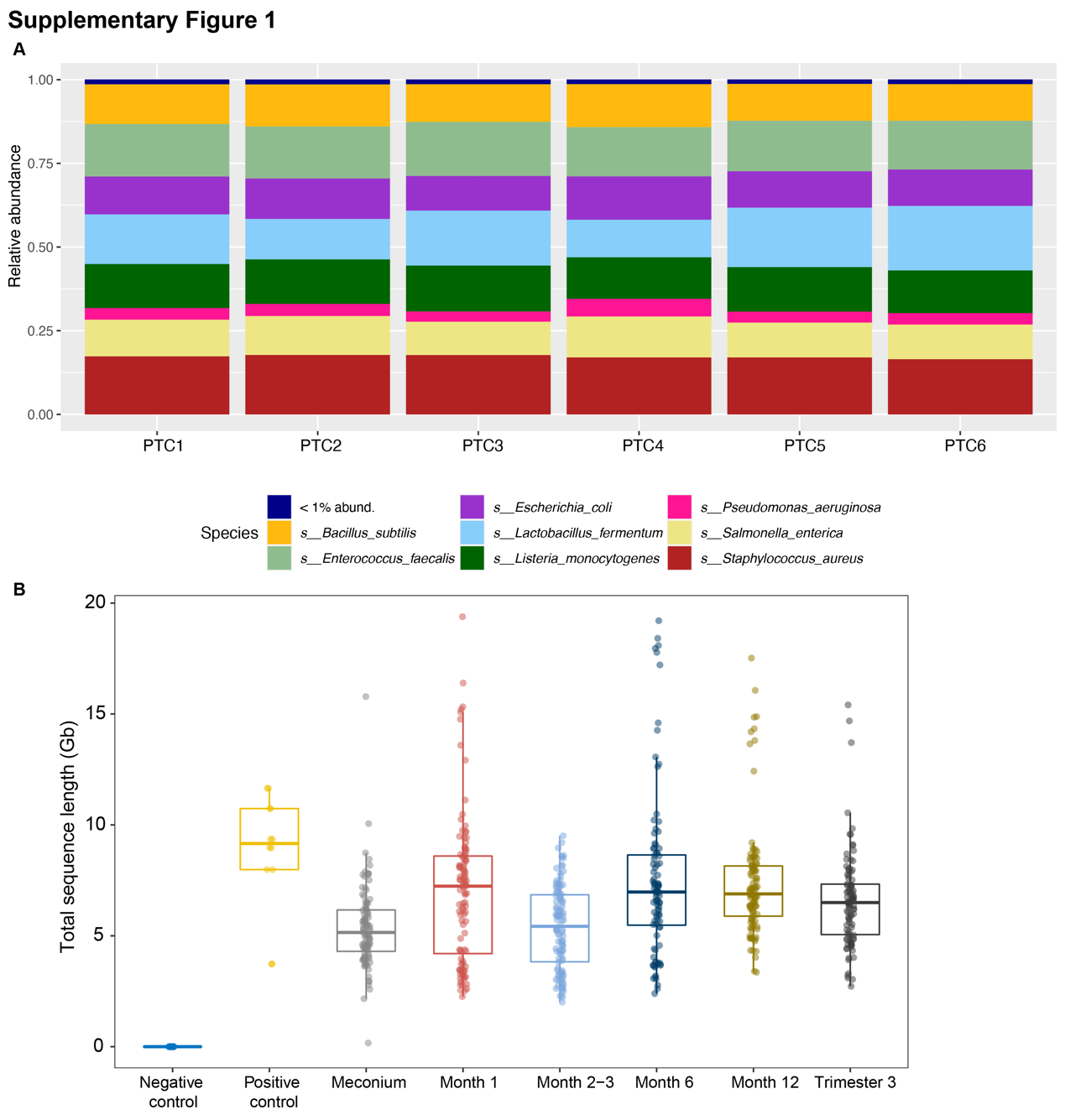
**

**Supplementary Figure 1. (A) Microbiota composition at the species level of positive control and (B) the sequence length of all samples.** For increased readability, Samples with a total sequence length higher than 20 Gb (n=4) have been removed in this figure.

**
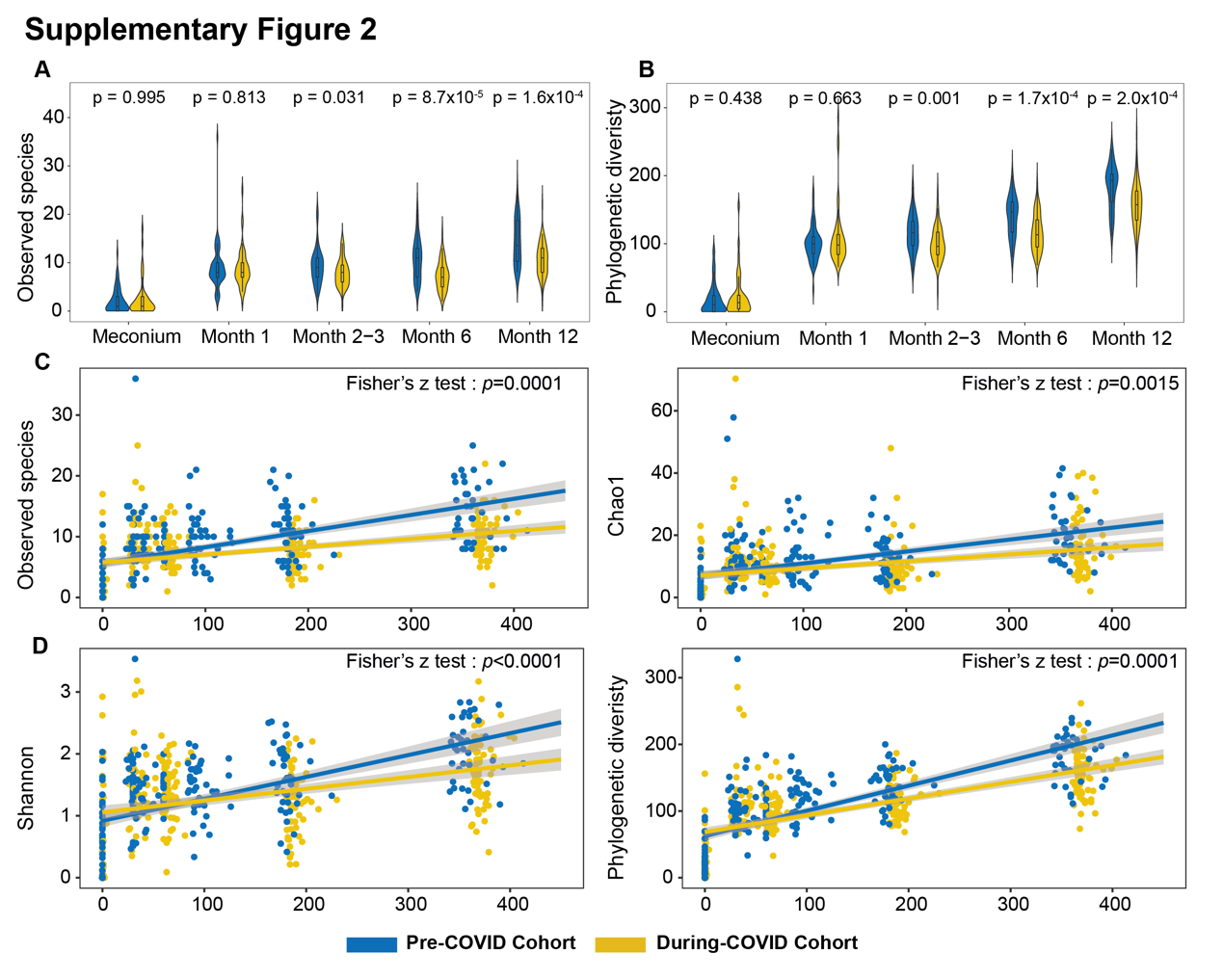
**

**Supplementary Figure 2. Gut microbial alpha diversity differed between infants pre-COVID and during-COVID pandemic within the first year of life. (A)** Microbial richness was assessed by the Observed index and **(B)** microbial diversity was assessed by Phylogenetic index. *P* values were given by Wilcoxon’s rank-sum tests. **(C)** The increasing rate of microbial richness and **(D)** diversity was higher in pre-COVID cohort than in during-COVID cohort. *P* values were given by Fisher's Z Transformation after converting Kendall’s Tau to Pearson’s r.

**
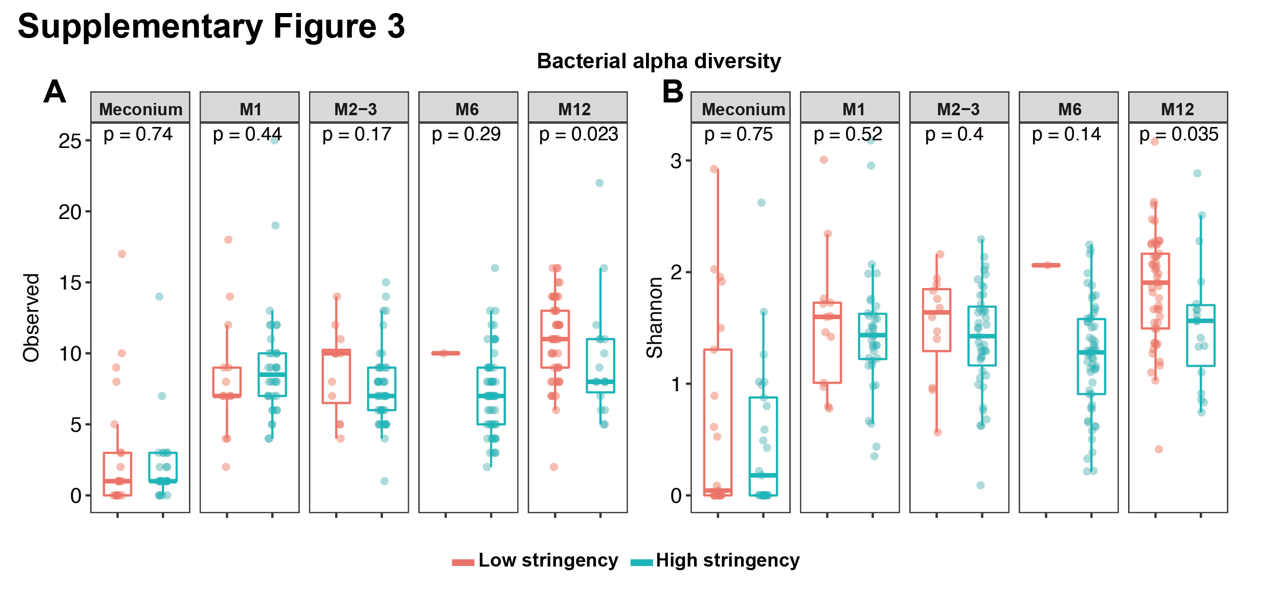
**

**Supplementary Figure 3. Differences in gut microbial alpha diversity during COVID-19 containment measures.** Samples in during-COVID group were further divided into high stringency and low stringency subgroups based on whether the samples were collected under control measures with a stringency level higher than three.

**
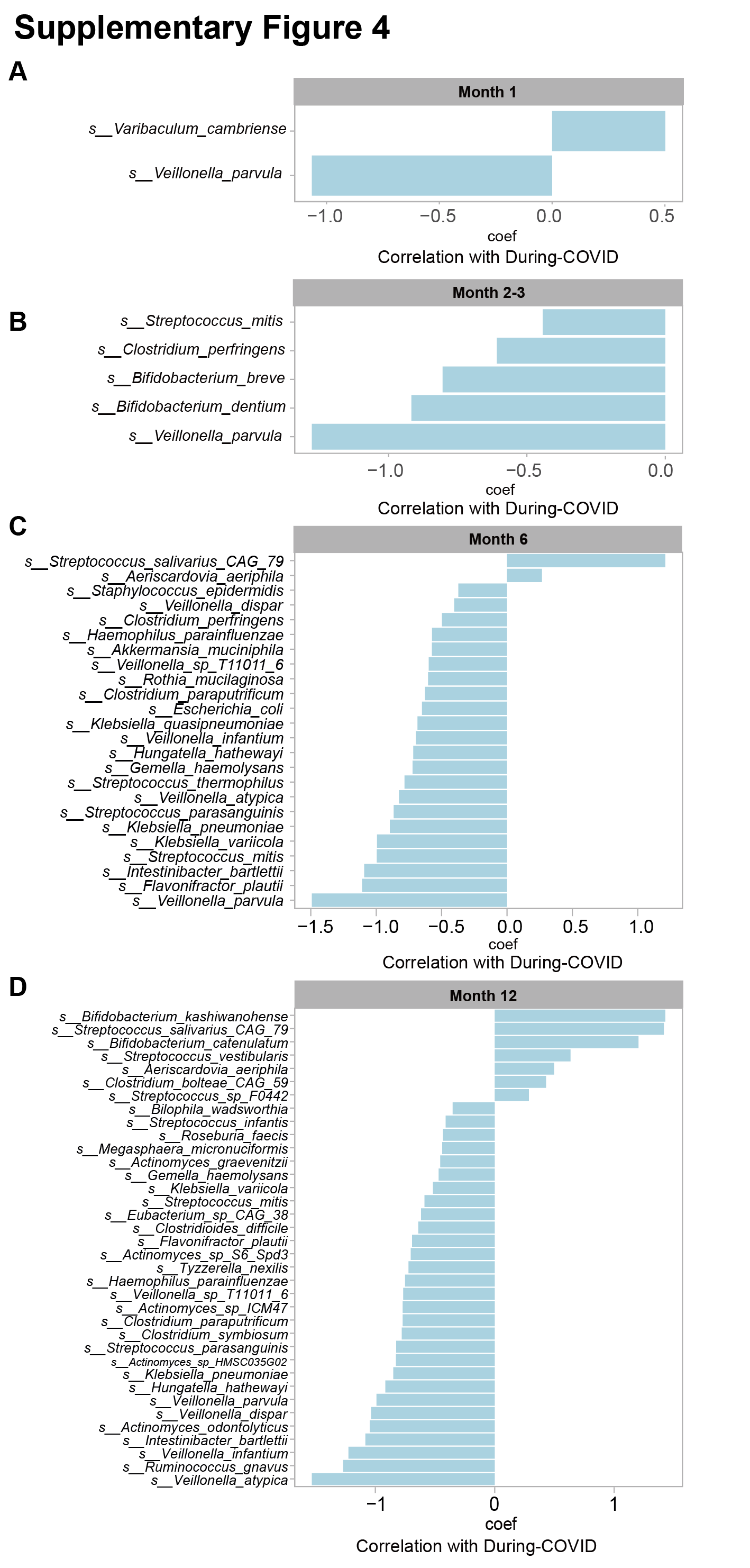
**

**Supplementary Figure 4 Differential bacterial species between pre-COVID and during-COVID cohort.** Differential bacterial species were identified by MaAsLin adjusted with delivery mode, intrapartum antibiotics usage, and furry pets at the age of **(A)** 1 month, **(B)** 2-3 months, **(C)** 6 months, and **(D)** 12 months.


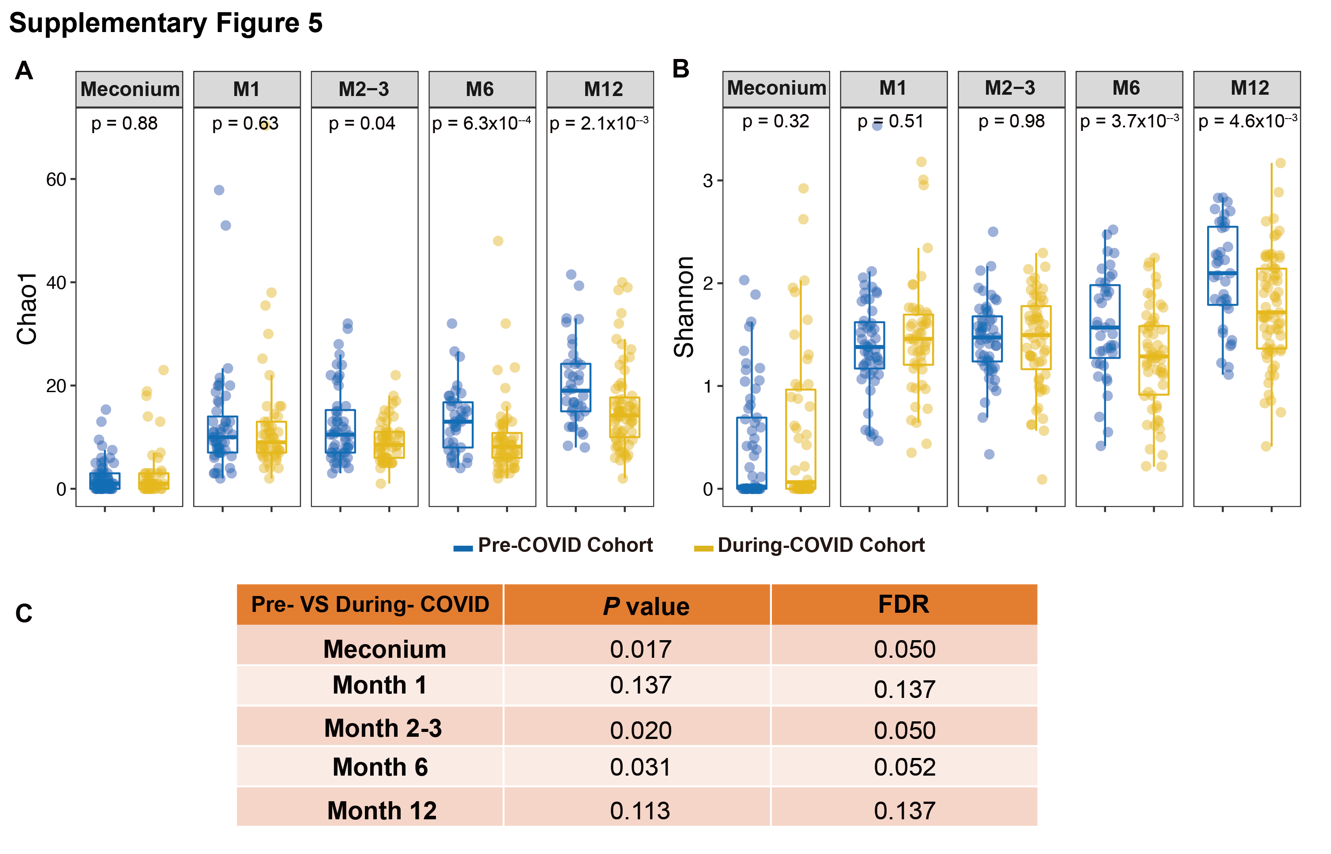


**Supplementary Figure 5 Sensitivity analysis on gut microbiota diversity and composition.** A sensitivity analysis that excluded the infants who were exposed to antibiotics was performed to investigate the difference in gut microbiota alpha diversity, including **(A)** Chao1 index and **(B)** shannon index, and **(C)** composition between the pre-COVID cohort and during-COVID cohort.

**
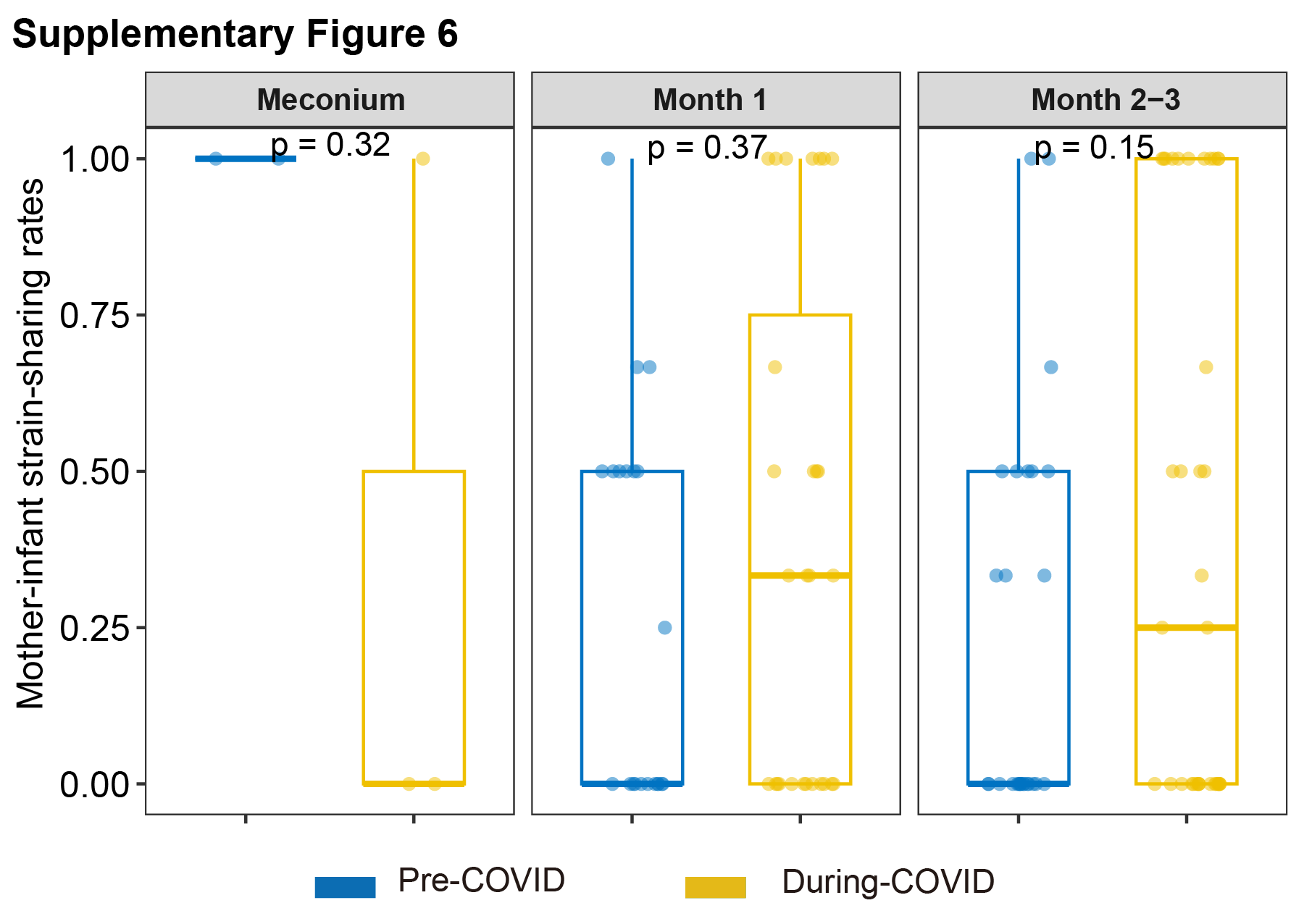
**

**Supplementary Figure 6 Difference in mother-infant microbial strain-sharing rate between pre-COVID cohort and during-COVID cohort.** Strain distance was defined as for PanPhlAn including normalization of each tree by its median value. A pair of strains with a strain distance lower than 0.1 was considered the same strain. Strain-sharing rates were calculated as the number of shared strains divided by the number of species common to each pair of individuals.

**
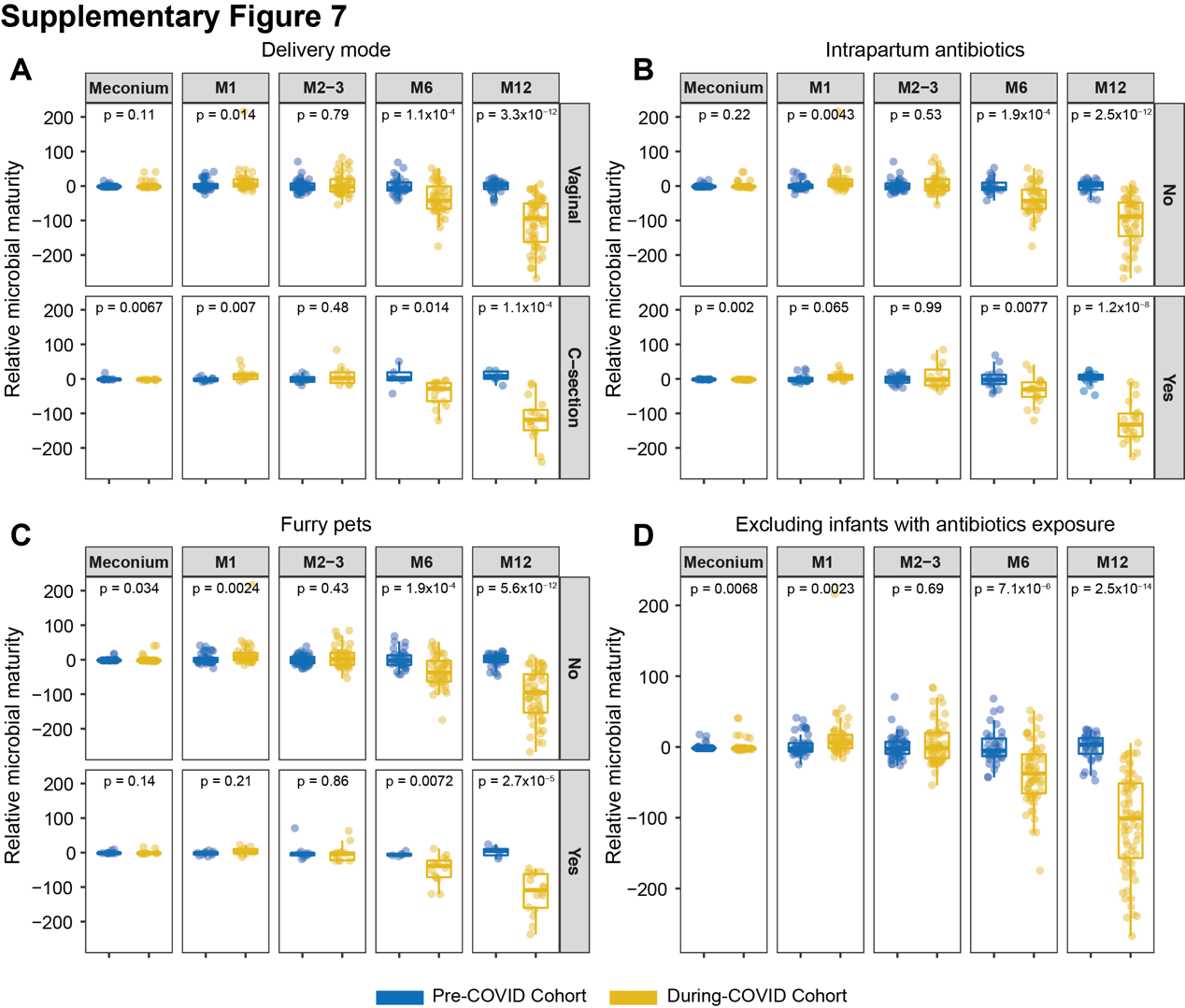
**

**Supplementary Figure 7 The relative microbial maturity in during-COVID cohort remained lower than that of the pre-cohort at 6 and 12 months of age after stratification by confounding factors.** The difference in relative microbial maturity between pre-COVID cohort and during-COVID cohort, stratified by **(A)** delivery mode, **(B)** intrapartum antibiotic exposure, and **(C)** the presence of household pets. **(D)**The difference in relative microbial maturity between pre-COVID cohort and during-COVID cohort after excluding those infants with antibiotic exposure.


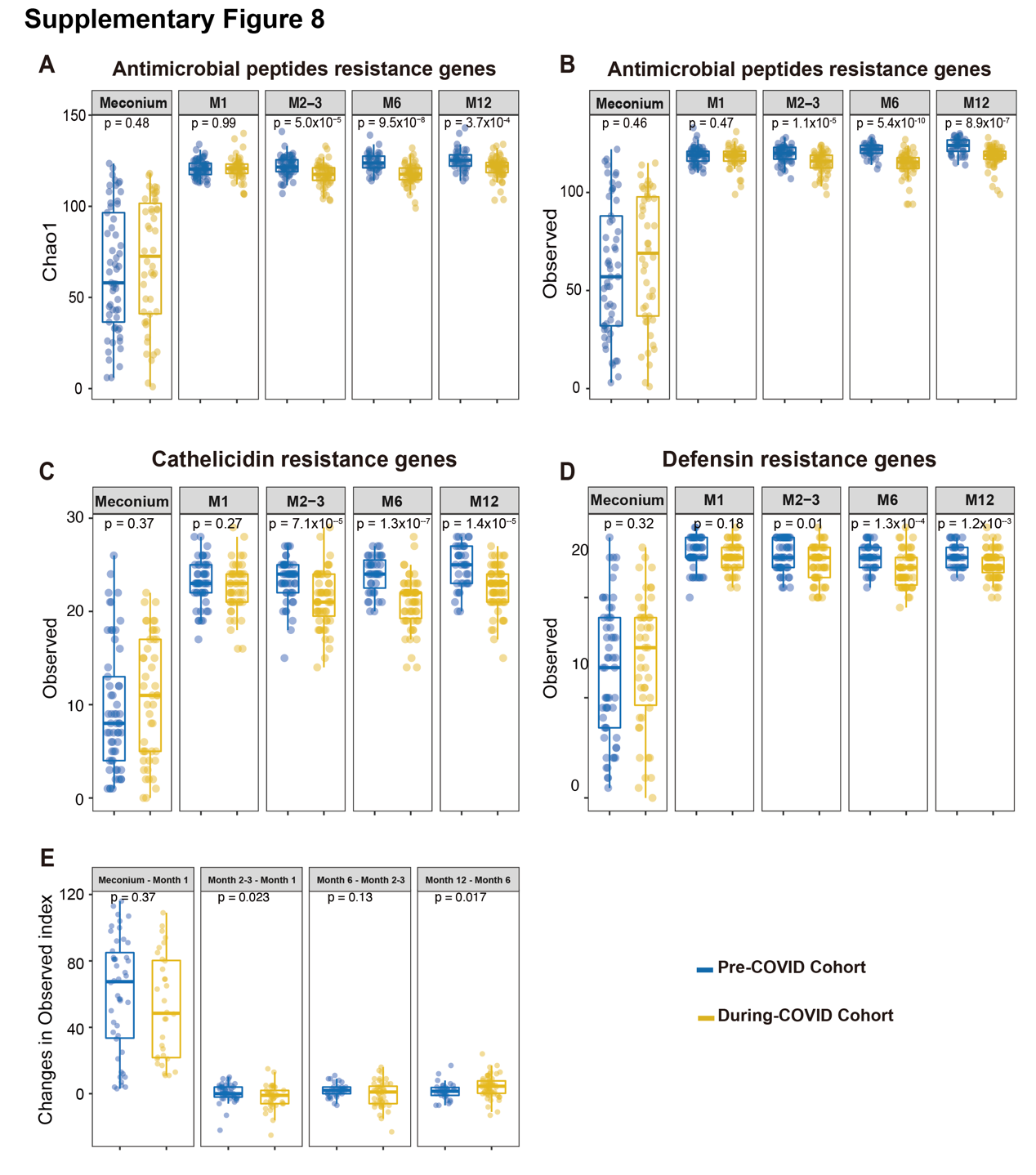


**Supplementary Figure 8 AMPs resistance genes reservoir differed between infants pre-COVID and during-COVID pandemic within the first year of life. (A)** Data represents Chao1 index of AMPs resistance genes in faecal sample. **(B)** Data represents Observed index of AMPs resistance genes in faecal sample. *P* values were given by Wilcoxon’s rank-sum tests. Sensitivity analysis on the richness of gut microbial **(C)** cathelicidin resistance genes and **(D)** defensin resistance genes. **(E)** The longitudinal patterns of AMP resistance genes acquisition differed between the pre-COVID cohort and the during-COVID cohort. P values were determined by Wilcoxon’s rank-sum tests.


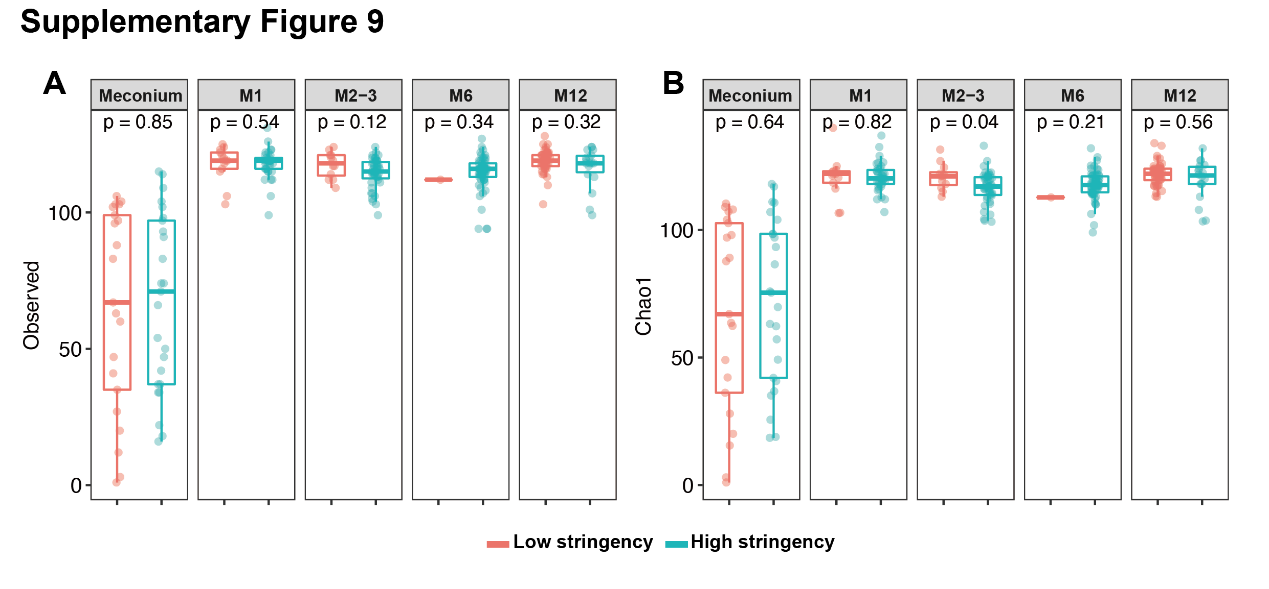


**Supplementary Figure 9 The richness of AMP resistance genes during COVID-19 containment measures.** Differences in the **(A)** observed index and **(B)** Chao1 index of AMP resistance genes between low and high stringency subgroup.


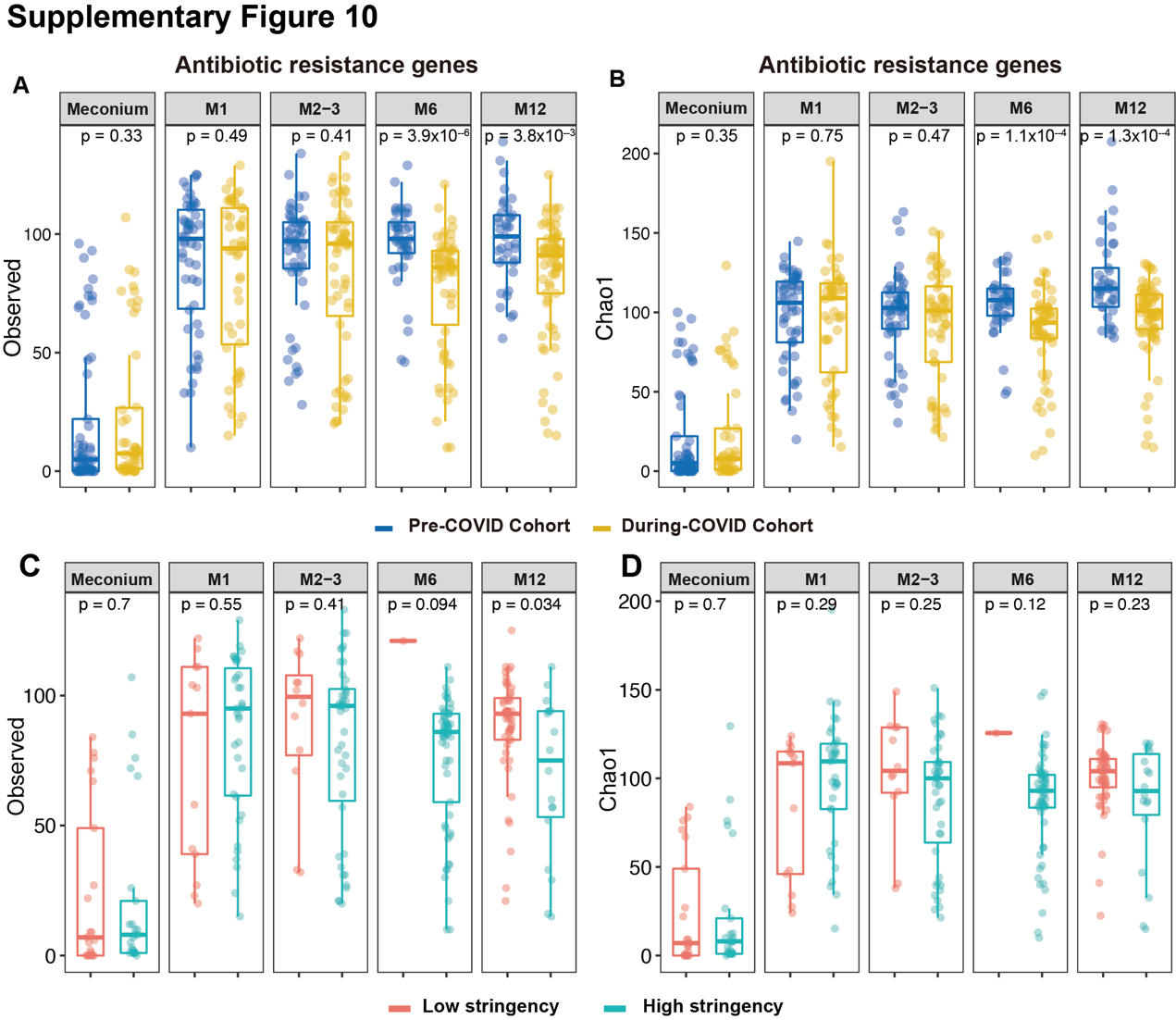


**Supplementary Figure 10 Sensitivity analysis on richness of gut microbial ARGs.** A sensitivity analysis that excluded the infants who were exposed to antibiotics was performed to investigate the difference in the **(A)** observed index and **(B)** the Chao1 index of antibiotics resistance genes. The richness of AMP resistance genes during COVID-19 containment measures. Differences in the **(C)** observed index and **(D)** Chao1 index of ARGs resistance genes between low and high stringency subgroup.

**Supplementary Table 1 Breastfeeding practice at the age of 6 and 12 months old**

|  | **Pre-COVID**  **(N=44)** | **During-COVID**  **(N=67)** | ***p*-value** |
| --- | --- | --- | --- |
| **M6 Breastfeeding practice** |  |  | 0.107 |
| **Almost exclusive breastfeeding^*^** | 9 (20.5%) | 16 (23.9%) |  |
| **Mixed feeding** | 15 (34.1%) | 11 (16.4%) |  |
| **Almost formula feeding+** | 20 (45.5%) | 39 (58.2%) |  |
| **Missing** | 0 | 1 |  |
| **M12 Breastfeeding practice** |  |  |  |
| **Almost exclusive breastfeeding^*^** | 8 (18.2%) | 6 (9.0%) | 0.06 |
| **Mixed feeding** | 7 (15.9%) | 4 (6.0%) |  |
| **Almost formula feeding+** | 29 (65.9%) | 57 (85.1%) |  |

Data are n (%) unless otherwise indicated

* Almost exclusive breastfeeding: the proportion of breastfeeding higher than 90%

+ Almost formula feeding: the proportion of formula feeding higher than 90%

**Supplementary Table 3 Characteristics of mothers between Pre-COVID and During-COVID group**

|  | **Pre-COVID (N=40)^+^** | **During-COVID (N=59)** | ***p*-value** |
| --- | --- | --- | --- |
| **Age** | 33.04 (4.0) | 32.17 (3.9) | 0.28 |
| **Ever smoking** | 6 (15) | 4 (6.8) | 0.42 |
| **Education** |  |  | 0.98 |
| **Bachelor degree or above** | 25 (62.5) | 37 (62.7%) |  |
| **High school or below** | 15 (37.5) | 22 (37.3) |  |
| **Gestational hypertension** | 3 (7.5) | 1 (1.7) | 0.3 |
| **Gestational diabetes mellitus** | 5 (12.5) | 5 (8.6) | 0.74 |

Data are n (%) for categorical variables and mean (SD) for continuous variable

^+^Above information was missing in 4 out of 44 mothers in pre-COVID cohort

**Supplementary Table 4 Transmission event of gut microbiota between mother and infant in pre-COVID cohort and during-COVID cohort**

|  | **pre-COVID (n=39)** | **During-COVID (n=46)** |  | **pre-COVID (n=33)** | **During-COVID (n=42)** |  | **pre-COVID (n=38)** | **During-COVID (n=50)** |  |
| --- | --- | --- | --- | --- | --- | --- | --- | --- | --- |
|  | **Meconium** | | **p-value** | **M1** | | **p-value** | **M2-3** | | **p-value** |
| ***Bacteroides_fragilis*** | 0 | 0 | 1 | 6 | 4 | 0.32 | 6 | 3 | 0.17 |
| ***Bifidobacterium_bifidum*** | 0 | 0 | 1 | 0 | 3 | 0.25 | 0 | 4 | 0.13 |
| ***Bifidobacterium_longum*** | 1 | 1 | 1 | 4 | 10 | 0.24 | 6 | 10 | 0.61 |
| ***Clostridium_sp_CAG_242*** | 0 | 0 | 1 | 0 | 1 | 1 | 0 | 0 | 1 |
| ***Escherichia_coli*** | 1 | 0 | 0.46 | 1 | 0 | 0.44 | 1 | 0 | 0.43 |
| ***Klebsiella_pneumoniae*** | 0 | 0 | 1 | 0 | 0 | 1 | 0 | 1 | 1 |
| ***Lactobacillus_salivarius*** | 0 | 0 | 1 | 0 | 1 | 1 | 0 | 1 | 1 |
| ***Parabacteroides_distasonis*** | 1 | 0 | 0.46 | 3 | 0 | 0.08 | 2 | 0 | 0.18 |
| ***Ruminococcus_lactaris*** | 0 | 0 | 1 | 0 | 2 | 0.5 | 0 | 0 | 1 |
| ***Streptococcus_salivarius*** | 0 | 0 | 1 | 0 | 1 | 1 | 0 | 2 | 0.5 |

n indicates mother-baby pairs

p-value was calculated the Chi-square test or Fisher’s exact test

**Supplementary Table 7 Sensitivity analysis of AMP/ARGs resistance genes in specific bacteria between Pre-COVID and During-COVID excluding infants exposed to antibiotics in the first year**

| **Category** | **Species** | **Median (Pre-COVID)** | **Median (During-COVID)** | **Mean (Pre-COVID)** | **Mean (During-COVID)** | **Effect size** | **p-value** |
| --- | --- | --- | --- | --- | --- | --- | --- |
| Cathelicidin | *Enterococcus faecalis* | 2 | 2 | 1.07 | 1.52 | 0.239 | 0.017 |
| Cathelicidin | *Staphylococcus epidermidis* | 2 | 2 | 2 | 1.55 | 0.417 | 0.016 |
| Cathelicidin | *Staphylococcus aureus* | 9 | 8.5 | 9 | 7.56 | 0.494 | 0.01 |
| Defensin | *Staphylococcus epidermidis* | 2 | 2 | 2 | 1.55 | 0.417 | 0.016 |
| Defensin | *Staphylococcus aureus* | 11 | 10 | 10.7 | 8.89 | 0.346 | 0.071 |
| Polymyxin | *Staphylococcus epidermidis* | 2 | 2 | 2 | 1.5 | 0.492 | 0.004 |
| Polymyxin | *Staphylococcus aureus* | 3 | 3 | 3 | 2.33 | 0.379 | 0.048 |
| Fluoroquinolone | *Staphylococcus epidermidis* | 3 | 2 | 2.57 | 1.8 | 0.533 | 0.002 |
| Fluoroquinolone | *Klebsiella pneumoniae* | 10 | 10 | 8.86 | 9.61 | 0.274 | 0.023 |
| Beta-Lactams | *Staphylococcus epidermidis* | 2 | 1.5 | 2.07 | 1.5 | 0.354 | 0.041 |
| Rifamycin | *Bifidobacterium breve* | 1 | 0 | 0.68 | 0.3 | 0.369 | 0.008 |

Effect size and statistical significance were determined via Wilcoxon rank-sum test
